# Schwann cell nodal membrane disruption triggers bystander axonal degeneration in a Guillain-Barré syndrome mouse model

**DOI:** 10.1172/JCI158524

**Published:** 2022-07-15

**Authors:** Rhona McGonigal, Clare I. Campbell, Jennifer A. Barrie, Denggao Yao, Madeleine E. Cunningham, Colin L. Crawford, Simon Rinaldi, Edward G. Rowan, Hugh J. Willison

**Affiliations:** 1 Institute of Infection, Immunity & Inflammation, University of Glasgow, Glasgow, United Kingdom.; 2 Nuffield Department of Clinical Neurosciences, University of Oxford, Oxford, United Kingdom.; 3 SIBPS, University of Strathclyde, Glasgow, United Kingdom.

**Keywords:** Autoimmunity, Neuroscience, Demyelinating disorders

## Abstract

In Guillain-Barré syndrome (GBS), both axonal and demyelinating variants can be mediated by complement-fixing anti–GM1 ganglioside autoantibodies that target peripheral nerve axonal and Schwann cell (SC) membranes, respectively. Critically, the extent of axonal degeneration in both variants dictates long-term outcome. The differing pathomechanisms underlying direct axonal injury and the secondary bystander axonal degeneration following SC injury are unresolved. To investigate this, we generated glycosyltransferase-disrupted transgenic mice that express GM1 ganglioside either exclusively in neurons [*GalNAcT^–/–^-Tg(neuronal)*] or glia [*GalNAcT^–/–^-Tg(glial)*], thereby allowing anti-GM1 antibodies to solely target GM1 in either axonal or SC membranes, respectively. Myelinated-axon integrity in distal motor nerves was studied in transgenic mice exposed to anti-GM1 antibody and complement in ex vivo and in vivo injury paradigms. Axonal targeting induced catastrophic acute axonal disruption, as expected. When mice with GM1 in SC membranes were targeted, acute disruption of perisynaptic glia and SC membranes at nodes of Ranvier (NoRs) occurred. Following glial injury, axonal disruption at NoRs also developed subacutely, progressing to secondary axonal degeneration. These models differentiate the distinctly different axonopathic pathways under axonal and glial membrane targeting conditions, and provide insights into primary and secondary axonal injury, currently a major unsolved area in GBS research.

## Graphical abstract

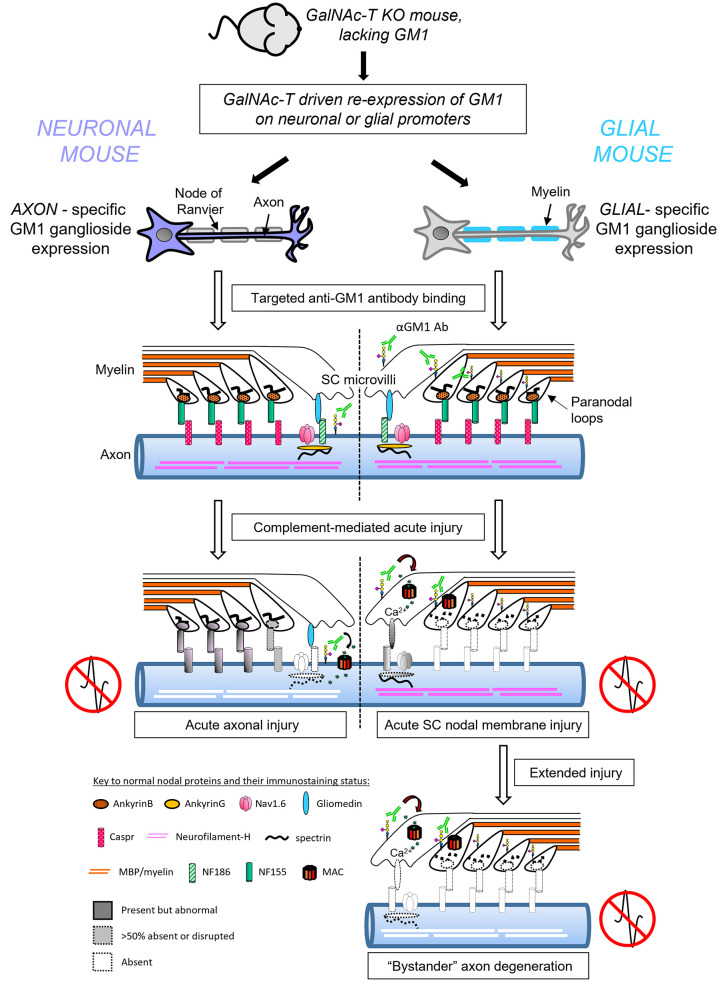

## Introduction

Guillain-Barré syndrome (GBS) is a postinfectious autoimmune disorder affecting the peripheral nervous system (PNS) resulting in acute-onset paralysis (1). GBS comprises a spectrum of axonal and demyelinating variants that exhibit distinct pathophysiological features. The 2 major variants are acute motor axonal neuropathy (AMAN) and acute inflammatory demyelinating polyneuropathy (AIDP), a classification derived from electrophysiological and morphological features and characterized by axonal injury and segmental demyelination, respectively. AMAN cases display either reversible conduction failure followed by rapid recovery, understood to be a consequence of axonal conduction block without axonal transection at the nodes of Ranvier (NoRs) and distal motor nerves (2, 3), or a severe outcome associated with inexcitable nerves and extensive proximal and distal axon degeneration (4). In contrast, AIDP cases exhibit conduction slowing and/or conduction block, normally considered to be associated with segmental demyelination, and usually fully recover following regeneration of the myelinating Schwann cell (SC). Recent electrophysiological data have highlighted the limitations of the AMAN/AIDP classification by introducing the alternative term “nodo-paranodopathy” to describe clinical situations in which either axonal or SC nodal membrane injury (or both) account for the acute conduction failure, in the absence of segmental demyelination as an early feature (3). The direct pathological evidence of this electrophysiological conceptualization is incomplete, and the clinical outcome varied. Apart from primary axonal injury, secondary bystander axonal degeneration may occur following SC membrane injury, either locally at the nodal region or with segmental demyelination. The long-term severity of GBS is dictated by the extent of the primary and/or secondary bystander axonal degeneration, supported by studies indicating that high serum levels of the axonal structural protein, neurofilament-light chain, can be predictive of poorer patient outcome (5). As such it remains critical to prognostic modeling and therapy development to differentiate and understand the pathological and degenerative pathways in action at the axo-glial interface in GBS, especially the mechanisms underlying secondary axonal degeneration in AIDP- and SC-restricted forms of GBS.

**Figure 1:**
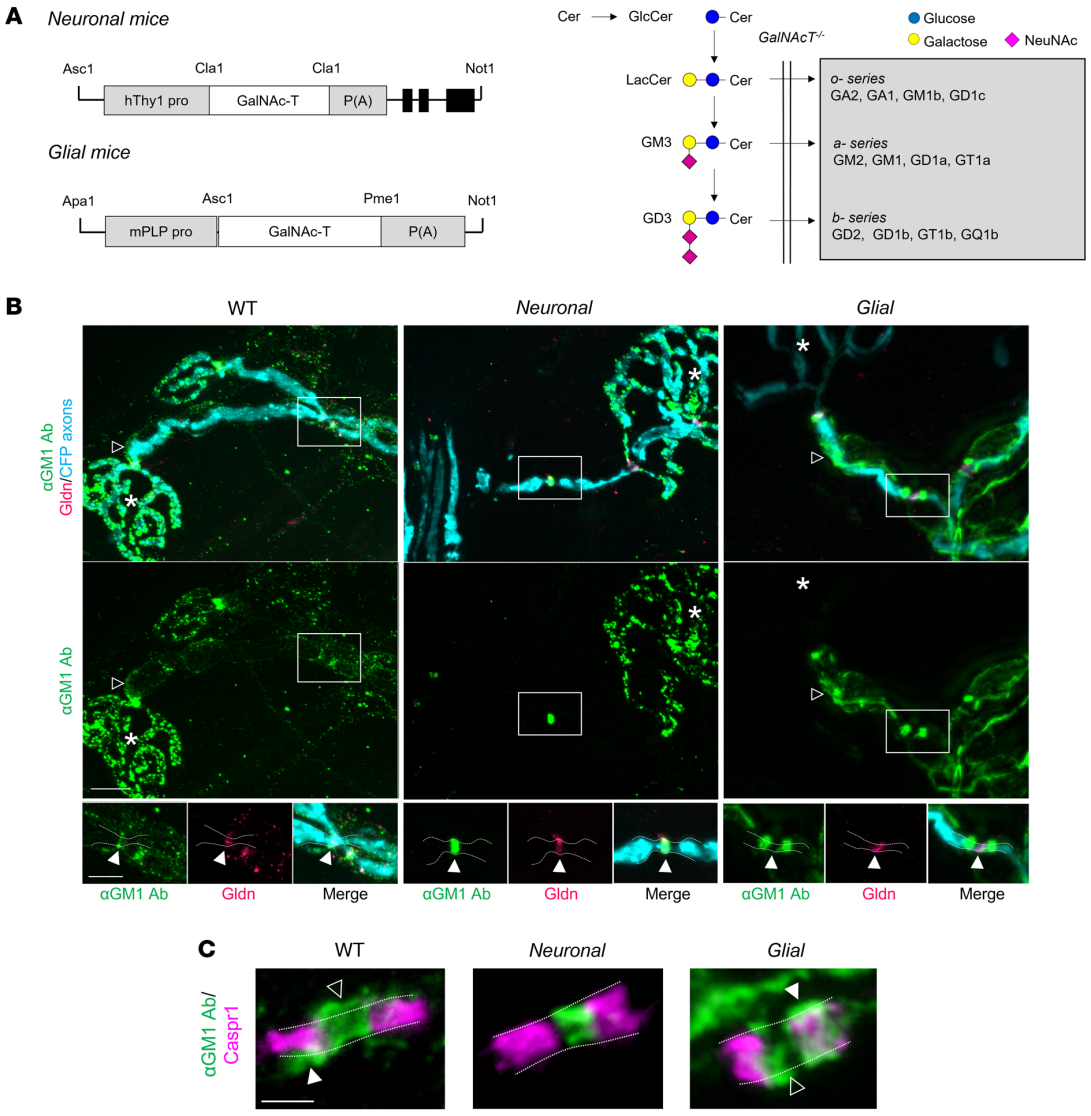
Anti–GM1 ganglioside antibody binding in transgenic mice with selective neuronal or glial complex ganglioside expression. (**A**) Constructs used to direct GalNAc-T expression in neurons (human *Thy1.2* promoter) or glia (mouse *Plp* promoter) of *GalNAc-T^–/–^-Tg(neuronal)* (*Neuronal*) and *GalNAc-T^–/–^-Tg(glial)* (*Glial*) mice, respectively. Ganglioside biosynthesis pathway indicates the reexpression of complex ganglioside expression (gray box) following construct insertion on a *GalNAc-T^–/–^* background (20). (**B**) Using a single anti-GM1 antibody (Ab, green), differential binding was observed at the distal motor nerves from triangularis sterni nerve–muscle preparations among genotypes. Open arrowheads indicate internodal Schwann cell (SC) abaxonal membrane anti-GM1 Ab deposition on WT and *Glial* nerves (absent along *Neuronal* nerves). Gliomedin (Gldn) immunostaining identifies the nodal gap. Boxed areas are enlarged underneath and represent differential anti-GM1 Ab binding at nodes of Ranvier (NoRs) among genotypes in relation to gliomedin (closed arrowheads). Dashed lines delineate the border of the axonal membrane determined by cytoplasmic CFP–positive axons. Scale bars: 10 μm (top panels) and 5 μm (lower panels). Asterisks indicate motor nerve terminals. (**C**) Caspr1 immunostaining (magenta) indicates the paranodes. Dashed lines delineate the border of the axonal membrane and arrowheads indicate anti-GM1 Ab binding beyond this membrane, suggesting binding on the glial membranes of the SC microvilli (open arrowheads) or paranodal loops (closed arrowheads) at WT and *Glial* NoRs. Scale bar: 2 μm.

Evidence from patient serology and human postmortem nerve material indicates that autoantibody and complement system involvement with macrophage recruitment are prominent mediators of GBS (6–8). The best-understood antigens that underlie the postinfectious autoimmune response in GBS are glycans borne by bacterial lipo-oligosaccharides (LOS), notably from *Campylobacter jejuni*, that are structural mimics of neural gangliosides (9) (Figure 1A). The predominant neural ganglioside and LOS mimic is the oligosaccharide of GM1. Anti-GM1 IgG antibodies occur most frequently in AMAN, but also occur, albeit less commonly, in GBS cases classified as AIDP; the explanation(s) differentiating the clinical presentation of AMAN or AIDP are unknown. Additionally, anti-GM1 antibodies can lead to either reversible conduction failure or axonal degeneration in AMAN patients (10).

Gangliosides are highly enriched in nervous system plasma membranes relative to other tissues (11). GM1 is expressed on both axolemmal and SC membranes and is particularly exposed at NoRs and motor nerve terminals (MNTs) (12), sites essential for saltatory conduction and synaptic transmission, respectively. The development of transgenic mice null for β4-GalNAc transferase that synthesizes complex gangliosides has demonstrated their essential role in the stability and function of the NoR (13). Significantly, the NoR is a major site of pathophysiology in GBS, first established from autopsy material that showed a lengthening of the nodal gap and axonal pathology with nodal autoantibody and complement deposition (6, 7). Subsequently, an anti-GM1 antibody–driven rabbit model of AMAN replicated this nodal lengthening (14). Combined with rodent models, a causative role for anti-ganglioside antibody (AGAb) activation of the complement cascade leading to dysfunction of the nerve was thus established (14–16). These models demonstrated loss of axonal integrity, eversion of paranodal loops, and disruption of key axo-glial adhesion proteins: contactin-associated protein 1 (Caspr1) localization on the axonal membrane and neurofascin 155 (NF155) on the glial membrane, that together contribute to the formation of the normal paranodal axo-glial junction. Significantly, ankyrin G (AnkG), the axonal anchoring protein responsible for tethering and maintaining voltage-gated sodium (Nav) channels in conjunction with neurofascin 186 (NF186) to the actin-spectrin cytoskeleton at the NoR (17), was also disturbed in these models. Later, the direct axonopathic mechanism was shown to be mediated through membrane attack complex (MAC) pore formation, calcium influx (18), and activation of the calcium-dependent protease calpain (16, 19) for which neurofilament, AnkG, actin, and spectrin are substrates.

Since gangliosides, including GM1, are expressed on both neuronal and glial membranes, it is impossible to separate primary and secondary injury from the consequences of a cell-specific (axonal or glial) membrane injury. Understanding these pathway differences is a crucial step in understanding the diverse degenerative mechanisms and informing the development of GBS variant–specific models and therapeutics. To unravel this, we developed transgenic mice that express gangliosides, including GM1, selectively on either glial or neuronal membranes (20). Here, we delineate axonal fate in distal motor nerve NoRs and nerve terminals, consequent to selective targeting of either SC or axonal membranes in these mice.

## Results

### Site-directed targeting of complex ganglioside expression to distal motor nerve membranes.

In order to exclusively target axonal or glial membranes via anti-GM1 antibody–mediated injury, we developed transgenic mouse strains that have complex ganglioside expression limited to neurons (and thus also their axon projections) through the human *Thy1.2* promoter [*GalNAc-T^–/–^-Tg(neuronal)*, *Neuronal*] or myelinating and non-myelinating glia through the mouse proteolipid protein (*Plp*) promoter [*GalNAc-T^–/–^-Tg(glial)*, *Glial*] (Figure 1A), as previously reported (12, 20, 21). Firstly, we used triangularis sterni (TS) nerve–muscle preparations to study the differing binding patterns of a single monoclonal anti-GM1 antibody (DG2) (22) at distal nerves and NoRs from *Neuronal* and *Glial* mice compared to wild-type (WT) mice (Figure 1, B and C). In WT nerve, anti-GM1 antibody binding was observed on the axonal membrane at the nerve terminal and weakly along the SC abaxonal membrane of the internode (open arrowheads, Figure 1B). Staining was enriched at the NoRs and colocalized with the SC microvilli marker gliomedin, and with paranodal Caspr1 at the node-proximal paranode. Binding on the glial SC microvilli and paranodal loop membranes was indicated by immunostaining beyond the border of axonally expressed cyan fluorescent protein (CFP) and Caspr1, demarcated by dashed lines and arrowheads in Figure 1, B and C. In *Neuronal* distal motor nerve, binding was restricted to the axolemma at the nerve terminal and NoRs; specifically, it colocalized with nodal gliomedin, was found between Caspr1 domains, was not detected beyond the demarcated axonal membrane, and was not observed anywhere along the SC abaxonal membrane. In *Glial* mice, internodal SC abaxonal membranes were positive, with the greatest enrichment at the paranodal loops most proximal to the nodal gap (node-proximal paranodal loops), flanking gliomedin. In some cases, staining could also be observed between Caspr1 domains and beyond the axonal membrane, which suggests SC microvilli positivity. Deposition on the axolemma was not clearly detected at the terminal or NoRs (also see Supplemental Figure 1A; supplemental material available online with this article; https://doi.org/10.1172/JCI158524DS1). A similar pattern for all genotypes was observed with cholera toxin B subunit labeling (a GM1 ligand, data not shown). As nerve terminal injury has previously been reported using *Neuronal* mice (21), the current research focuses on characterizing nerve terminal and NoR injury induced in *Glial* mice and the subsequent effects on axonal integrity.

### Distal motor nerve axonal integrity remains intact in an acute ex vivo model following selective glial targeting.

**Figure 2:**
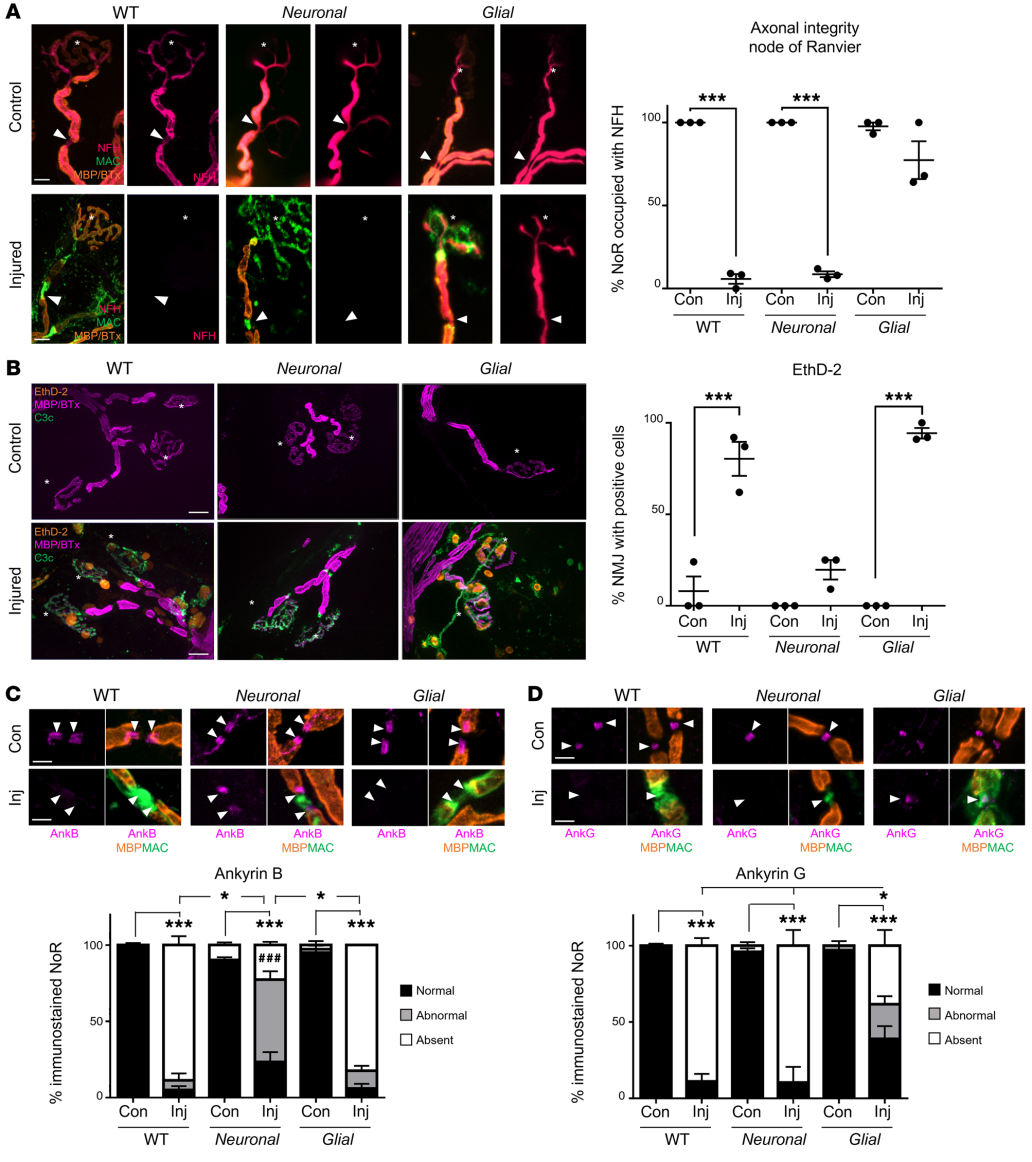
Distal motor nerve integrity following selective targeting and acute injury of neural membranes ex vivo. Triangularis sterni nerve–muscle preparations from WT, *Neuronal*, and *Glial* mice were treated ex vivo with anti-GM1 Ab and a source of complement (injury, Inj) or anti-GM1 Ab alone (control, Con). (**A**) Loss of axonal integrity due to injury at the motor nerve terminal (MNT, identified by α-bungarotoxin, BTx, orange, asterisk) and node of Ranvier (NoR, orange, arrowheads) was monitored by presence of neurofilament H immunostaining (NFH, magenta). Membrane attack complex (MAC) complement pore deposition (green) was present in all injured preparations compared with control. (**B**) Ethidium homodimer–positive (EthD-2–positive, orange) cells overlying MNT (magenta, asterisk) were compared among treatment groups. Representative images show the presence of complement deposition (green) in all injured tissue. (**C** and **D**) The sites where ankyrin B (AnkB) or AnkG immunostaining should be located are indicated by arrowheads. The presence of normal (black bars, statistical comparisons indicated with asterisks) or abnormal (gray bars) AnkB and AnkG immunostaining was compared to associated controls for each genotype. A lengthened gap between AnkB domains is shown in a representative image from injured *Neuronal* tissue. Weakened, uneven AnkG staining in injured *Glial* tissue is shown in the representative image. Scale bars: 20 μm (**A**), 50 μm (**B**), and 5 μm (**C** and **D**). Results are represented as the mean ± SEM. *n =* 3/genotype/treatment: 10–46 NoRs/mouse (median = 21, NFH); 11–29 neuromuscular junctions (NMJs)/mouse (median *=* 18, EthD-2); 10–26 NoRs/mouse (median = 23, AnkG); and 12–31 NoRs/mouse (median *=* 21, AnkB) were analyzed. *P < 0.05; ***P < 0.001; ^###^*P* < 0.001 (for abnormal AnkB and AnkG immunostaining in **C**) compared with control by 2-way ANOVA with Tukey’s post hoc test.

The TS ex vivo complement-mediated nerve-muscle injury model was used to assess axonal integrity when selectively targeting glial membranes in *Glial* mice compared with WT and *Neuronal* mice. Our previous research has shown that complement deposition shares a similar binding pattern to AGAb, indicating site-specific antibody–directed targeting of complement deposits (15). When a source of complement (provided in the form of normal human serum, NHS) was added to preparations pretreated with anti-GM1 antibody (injury), MAC deposition was observed in a neural membrane–selective pattern (Supplemental Figure 1B), as expected from the immunostaining patterns shown in Figure 1, B and C. Thus, in WT mice MAC was deposited at NoRs (arrowheads) and weakly over the MNT (asterisks) (Figure 2A). MAC staining was absent from anti-GM1 antibody–only control tissue. In *Neuronal* mice, MAC pore deposition was restricted to the axonal membrane and present at both the NoRs and MNTs in injured tissue. In contrast, in *Glial* tissue, MAC deposition was observed at the node-proximal paranodal loops, the perisynaptic SCs (pSCs) overlying the MNTs, and was more weakly seen along the internodal abaxonal SC membrane. Thus, the pattern of MAC deposition recapitulates the distribution of anti-GM1 antibody deposition, indicating that complement is being activated specifically at anti-GM1 antibody binding sites.

Controls from each genotype exposed only to anti-GM1 antibody (lacking NHS as a complement source) showed normal axonal integrity at NoRs and MNTs, judged by intact neurofilament immunostaining occupying these sites. In this acute injury (4 hours) paradigm, MAC pore deposition in *Glial* mice did not cause a significant loss of axonal integrity at either NoRs or MNTs compared to control (Figure 2A). As expected, and in diametric contrast to the *Glial* mice, complement deposition resulted in almost complete loss of axonal integrity at the NoRs and MNTs in injured WT and *Neuronal* tissue.

Previous studies have shown that AGAb can bind and injure pSCs overlying the nerve terminal but does not induce loss of terminal axon integrity in an acute injury model of 1-hour duration (23). We used the cell viability label, ethidium homodimer (EthD-2), to assess the health of pSCs (Figure 2B). EthD-2–positive cell number significantly increased over MNTs from injured *Glial* and WT tissue compared with *Neuronal* tissue (*P <* 0.001, two-way ANOVA). Injury to pSCs alone, without direct axonal targeting, was insufficient to cause loss of axonal integrity at the MNTs at this acute time point. Taken together, these data suggest that direct targeting of the axonal membrane is necessary for acute loss of axonal integrity.

### Disruption of intracellular cytoskeletal proteins at NoRs following selective glial and neuronal targeting ex vivo.

The consequence of selective membrane targeting for glial and axonal cytoskeletal anchoring protein localization at NoRs was comparatively analyzed ex vivo in TS preparations exposed to anti-GM1 antibody and complement. The glial paranodal cytoskeletal anchoring protein, ankyrin B (AnkB) (24), and the axonal cytoskeletal anchoring protein, AnkG, were compared at the NoRs in *Neuronal*, *Glial*, and WT tissue (Figure 2, C and D). When tissue was injured through the addition of NHS, AnkB immunostaining was lost in *Glial* and not *Neuronal* mice, although it appeared disrupted in the latter, with either weaker staining or a lengthened gap between domains, signifying either a lengthened nodal gap and/or a loss of NF155 from the innermost proximal-nodal border. Conversely, AnkG immunostaining was significantly absent from injured *Neuronal* compared with *Glial* NoRs (Figure 2D, *P <* 0.05, two-way ANOVA). A degree of disruption, as defined by an abnormal immunostaining pattern (outlined in Methods), to AnkB and AnkG occurred in *Neuronal* and *Glial* mice, respectively, indicating some subtle reciprocal disturbance of axoglial integrity when one or the other membrane is targeted. In WT mice in which both glial and axonal components of the NoR were targeted, both AnkB and AnkG were almost entirely absent.

### Disruption of axo-glial adhesion proteins at NoRs following selective glial and neuronal targeting ex vivo.

As AnkB and AnkG are key cytoskeletal proteins in the glial and axonal cytoplasmic compartments of NoRs, respectively, we next studied the disruption to the axo-glial cell adhesion molecules (CAMs: glial, NF155; axonal, NF186). A fraction of AnkB interacts with NF155 in the paranodal loops (24) and AnkG anchors NF186 to the cytoskeleton at the axonal nodal membrane (25). Caspr1 is the axonal partner of glial NF155 and together with contactin-1 these CAMs form the major component of the paranodal axo-glial junction.

**Figure 3:**
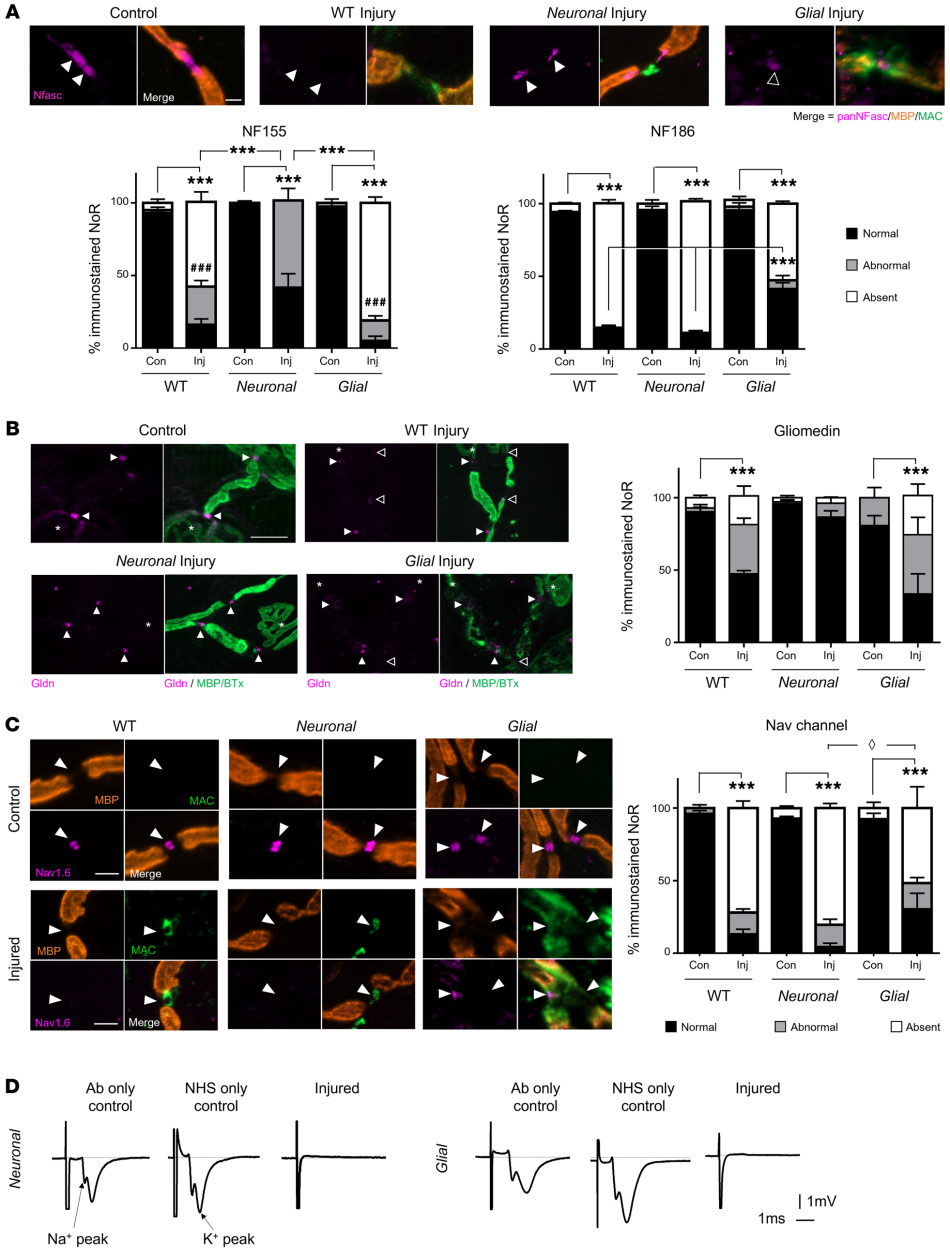
Differential disruption to the node of Ranvier when neuronal and glial membranes are injured selectively ex vivo. Triangularis sterni nerve–muscle preparations from WT, *Neuronal*, and *Glial* mice were treated ex vivo with anti-GM1 Ab and a source of complement (injury, Inj) or anti-GM1 Ab alone (control, Con). Disruption to nodal protein (magenta) organization at the node of Ranvier (NoR) due to injury was assessed; the site of expected staining is indicated by arrowheads for each marker. Representative images demonstrate normal nodal protein localization in all control tissue and absent or abnormal staining in injury groups, which coincides with nodal complement deposition (**A** and **C**, green). (**A**) A pan-neurofascin (Nfasc) Ab was used to assess paranodal NF155 (closed arrowheads) and nodal NF186 (open arrowhead). (**B**) SC microvilli marker gliomedin (Gldn) immunostaining at NoRs was assessed compared to controls. Asterisks indicate motor nerve terminals. (**C**) Changes to normal (black bars) Nav1.6 labeling were observed in injured tissue from all genotypes compared with associated controls. Diamond defines statistical comparisons of absent immunostaining (white bars). (**D**) Perineural recordings from distal motor nerves were performed on tissue from *Neuronal* and *Glial* mice treated with anti-GM1 Ab only, a source of complement (normal human serum, NHS) only, or a combination of Ab and NHS (injured). Representative recordings from 1 mouse per treatment demonstrate that normal Na^+^ and K^+^ waveforms were lost when the tissue was injured. Scale bar: 5 μm. Results are represented as the mean ± SEM. *n =* 3/genotype/treatment: 13–36 NoRs/mouse (median *=* 24, pNFasc); 15–33 NoRs/mouse (median = 19, gliomedin); and 11–30 NoRs/mouse (median = 23, Nav1.6) were analyzed. *P < 0.05, **P < 0.01, ***P < 0.001 (for comparisons between normal immunostaining); ^###^*P* < 0.001 (for abnormal NF155 immunostaining in *Neuronal* injury group compared to WT or *Glial* imjury in **A**) compared with control by 2-way ANOVA with Tukey’s post hoc test.

A pan-neurofascin antibody (binds both NF155 and NF186) (26) was used to assess the integrity of glial NF155 and axonal NF186 in the different genotypes ex vivo (Figure 3A and Supplemental Figure 2A). After injury, paranodal NF155 immunostaining was absent from NoRs in *Glial* mice, but present in *Neuronal* mice NoRs, albeit disrupted. In contrast, nodal NF186 immunostaining was absent from NoRs in injured *Neuronal* and moderately preserved in injured *Glial* mice. In injured WT mice, immunostaining for both NF155 and NF186 was severely disrupted or lost entirely.

Given the alterations to NF186, we next studied its SC microvilli ligand gliomedin. Gliomedin immunostaining was present at the majority of NoRs across all genotypes and treatments, but with an abnormal distribution compared with control in injured WT and *Glial* mice (Figure 3B) demonstrating a disruption to the SC microvilli glial membrane when directly targeted. The less dramatic changes are consistent with the lower levels of anti-GM1 antibody deposits on SC microvilli relative to paranodal loops.

We next considered that an indirect effect on Caspr1, the axonal partner of NF155, might be evident when targeting the glial membranes (Supplemental Figure 2B). Results show that Caspr1 immunostaining was disrupted or lost in injured *Glial* and WT tissue in which AnkB and NF155 were also absent. In contrast, Caspr1 immunostaining was relatively preserved at injured *Neuronal* NoRs, but with similar abnormalities as described for NF155. We attribute this distinction between *Glial* and *Neuronal* Caspr1 integrity to the preservation of AnkB and NF155 in *Neuronal* mice. Intriguingly, despite disturbances to the paranodal axo-glial junction, in preliminary studies we did not detect an invasion of voltage-gated potassium (Kv1.1) channels into the paranodal domain, as may be expected (Supplemental Figure 2C). Instead, there appeared to be a lengthening of the NoR between the domains in injured tissue from all genotypes. Myelin basic protein (MBP, a marker for compact myelin) intensity measurements across the NoRs (Supplemental Figure 3A) showed a reduction at the lateral node-proximal edges in injured *Glial* and WT mice relative to *Neuronal* mice. This indicates disruption to the myelin at this site or a widening of the NoR by a reduction in intensity at the margins between the domains. Differences in MBP immunostaining patterns can be observed at NoRs throughout Figure 3. There were no measurable differences in internodal MBP intensity (data not shown), but staining was qualitatively abnormal in *Glial* mice, being punctate and vesiculated in the co-presence of MAC pore deposition, suggesting some early myelin damage (Supplemental Figure 3B). Additionally, EthD-2–positive SC nuclei were observed after acute injury in *Glial* preparations, indicating initial SC injury (Supplemental Figure 3C).

To firmly establish that the observed disruptions to paranodal proteins were not partly a confounding consequence of structural dysfunction secondary to abnormal ganglioside profiles in the axonal compartment of transgenic *Glial* mice, a complement-fixing antibody against the myelin glycolipid sulfatide (27) was used to target the paranodal loop glial membrane in WT mice in order to look for similar perturbations. As seen with the anti-GM1 antibody in *Glial* mice, AnkB and Caspr1 immunostaining was lost/abnormal compared with uninjured control (Supplemental Figure 4) at anti-sulfatide antibody–exposed paranodes.

### Disruption of axo-glial adhesion proteins by glial membrane targeting induces functional consequences similar to those of axonal targeting.

To assess the functional impact of disrupted anchoring proteins and CAMs ex vivo, we first studied Nav channel clustering by immunostaining (Figure 3C). All genotypes with NHS-induced injury had significantly fewer NoRs with normal Nav channel clustering compared with controls (*P <* 0.001, two-way ANOVA). Notably, there were significantly more NoRs with absent Nav channel clusters in injured *Neuronal* tissue compared with *Glial* tissue (*P <* 0.05, two-way ANOVA), suggesting a greater severity of injury to clustering when the axon is directly targeted; nevertheless, *Glial* injury also clearly affects Nav channel clustering, likely through an indirect pathway.

To study the consequent impact on function of losing Nav channel clustering and NoR architecture, we recorded perineural current waveforms from the distal nerves ex vivo (Figure 3D). In control observations, we recorded Na^+^ and K^+^ waveforms in *Glial* and *Neuronal* tissue under exposure to either anti-GM1 antibody alone or NHS alone. Perineural currents showed normal Na^+^ and K^+^ waveforms, as expected, and there were no changes in the Na^+^/K^+^ ratio among genotypes or treatments, showing no effect of antibody or NHS treatment alone (Supplemental Figure 3D). However, waveforms were completely absent from both injured *Glial* and *Neuronal* preparations, indicating severe functional impairment in both genotypes.

The above ex vivo results demonstrate that injury in WT leads to disruption of multiple proteins on both neuronal and glial membranes at the NoRs, as would be expected. However, selectively targeting the glial compartment principally causes SC nodal membrane disruption with mild axonal protein disruption, whereas targeting the axonal compartment principally leads to a major disruption of axonal structural and adhesion proteins, nodal lengthening, and leaves a partially intact axo-glial junction at the paranode. Significantly, either direct targeting to the axon or the SC nodal membrane has the same functional consequence: disruption to normal Nav channel clustering and acute conduction failure.

### Distal motor nerve axon integrity remains intact following acute selective glial membrane targeting in vivo.

Following the above characterization of the differential injury to the distal nerve in ex vivo models, we next sought evidence for selective injury in vivo. WT, *Neuronal*, and *Glial* mice were injected intraperitoneally (i.p.) with either anti-GM1 antibody and NHS (injury) or NHS only (control). The diaphragm was principally affected; therefore, noninvasive whole-body plethysmography was used to monitor respiratory function in live mice and the diaphragm was subsequently removed for immunostaining or morphological analysis.

**Figure 4:**
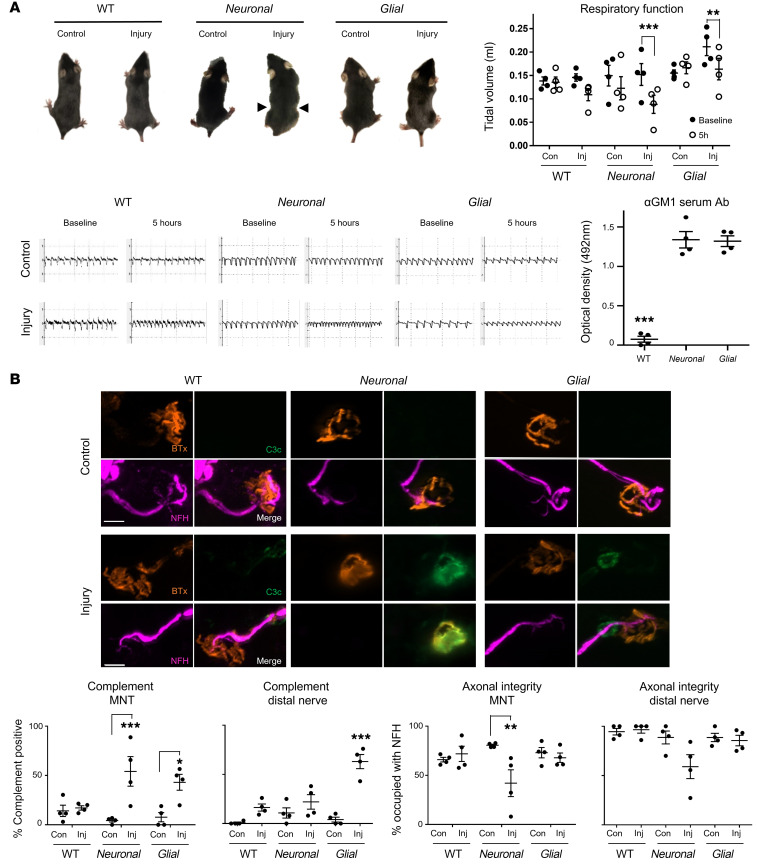
Distal motor nerve axonal integrity remains intact following selective glial membrane targeting in vivo. WT, *Neuronal*, and *Glial* mice were dosed i.p. with 50 mg/kg anti-GM1 Ab followed 16 hours later with 30 μL/g normal human serum (NHS) (injury, Inj) or NHS only (control, Con). Respiratory function was monitored and diaphragm distal nerves assessed by immunoanalysis 5 hours after NHS delivery. (**A**) Injured *Neuronal* mice displayed the most severe respiratory phenotype: a pinched, wasp-like abdomen (arrowheads) and significantly reduced tidal volume (TV) measured using whole-body plethysmography (EMMS). Injured *Glial* mice also had significantly reduced TV compared with baseline. Representative respiratory flow charts for each treatment group show reduced TV and an increase in respiratory rate. Serum analysis indicates that circulating anti-GM1 Ab could be detected in *Neuronal* and *Glial* but not WT mice. Results are represented as the mean ± SEM, *n =* 4/genotype/treatment. (**B**) Complement deposition and axonal integrity (neurofilament H [NFH] occupancy) were compared at the diaphragm motor nerve terminals (MNTs) and along distal nerves. Representative images illustrate complement deposits (green) overlying the MNT, identified by bungarotoxin (BTx, orange), in injured *Neuronal* mice, and on the distal nerve in injured WT and *Glial* mice. Scale bar: 10 μm. Results are represented as the mean ± SEM. *n =* 4/genotype/treatment: 68–133 MNTs/mouse (median = 103) and 7–30 NoRs/mouse (median *=* 15) were analyzed. *P < 0.05, **P < 0.01, ***P < 0.001 by repeated measures 2-way ANOVA with Bonferroni post-hoc tests (**A**) or 2-way ANOVA with Tukey post-hoc tests (**B**).

Injured *Neuronal* mice presented with the most severe respiratory phenotype consisting of a pinched, wasp-like abdomen indicative of a paralyzed diaphragm, as previously reported (21). In comparison, injured *Glial* and WT mice exhibited a milder respiratory dysfunction (Figure 4A). Tidal volume, used as a measure of respiratory function, was reduced at 5 hours after injury in all injured groups compared with baseline and genotype-matched control, as illustrated in representative respiratory flow charts, but most severely reduced in the *Neuronal* mice (Figure 4A). Additionally, the flow charts illustrate increased respiratory rate (tachypnoea) in all injured groups, although owing to a wide range in variability in individual mouse activity, this did not reach significance (data not shown). Serum from terminal bleeds was assessed for circulating anti-GM1 antibody and confirmed antibody presence in the *Neuronal* and *Glial* injury groups (Figure 4A) but was undetectable in WT mice. This finding replicates a phenomenon we previously reported where circulating antibody removal by endocytosis was observed in WT mice, thus rendering them unsuitable for in vivo injury modeling, as insufficient antibody remains available to bind and injure the intraneural target tissue (28).

We next assessed complement deposition and axonal integrity following in vivo injury in *Glial* mice compared to *Neuronal* mice (Figure 4B). MNTs with overlying complement deposits increased in injured *Glial* and *Neuronal* mice, reaching significance compared with their respective controls (*P <* 0.05 and *P <* 0.001, two-way ANOVA). Conversely, complement deposits were detected more readily along distal nerve SC membranes in injured *Glial* mice compared with all other treatment groups (*P <* 0.001, two-way ANOVA). Consistent with serum antibody results showing rapid AGAb sequestration, complement deposits were not detected to a significant degree compared to control in WT mice at either the MNTs or along the distal nerve. As seen in the acute ex vivo experiments, MNTs or distal nerves occupied by neurofilaments were not significantly lower in injured *Glial* mice compared to control, whereas injured *Neuronal* mice showed a significant reduction in neurofilament occupancy, and therefore axonal integrity (*P <* 0.01, two-way ANOVA). In WT mice, as expected from the absence of antibody and complement deposition, neurofilament occupancy was unchanged. These data thus show that complement deposition along the distal nerve in injured *Glial* mice does not affect axonal integrity in this acute injury paradigm, unlike injured *Neuronal* mice. Because of the lack of significant antibody and complement deposition in injured WT mice, and normal NoR conformation (Supplemental Figure 5A), they were not subjected to further in vivo studies.

### Disruption of structural and axo-glial adhesion proteins following glial membrane targeting in vivo.

**Figure 5:**
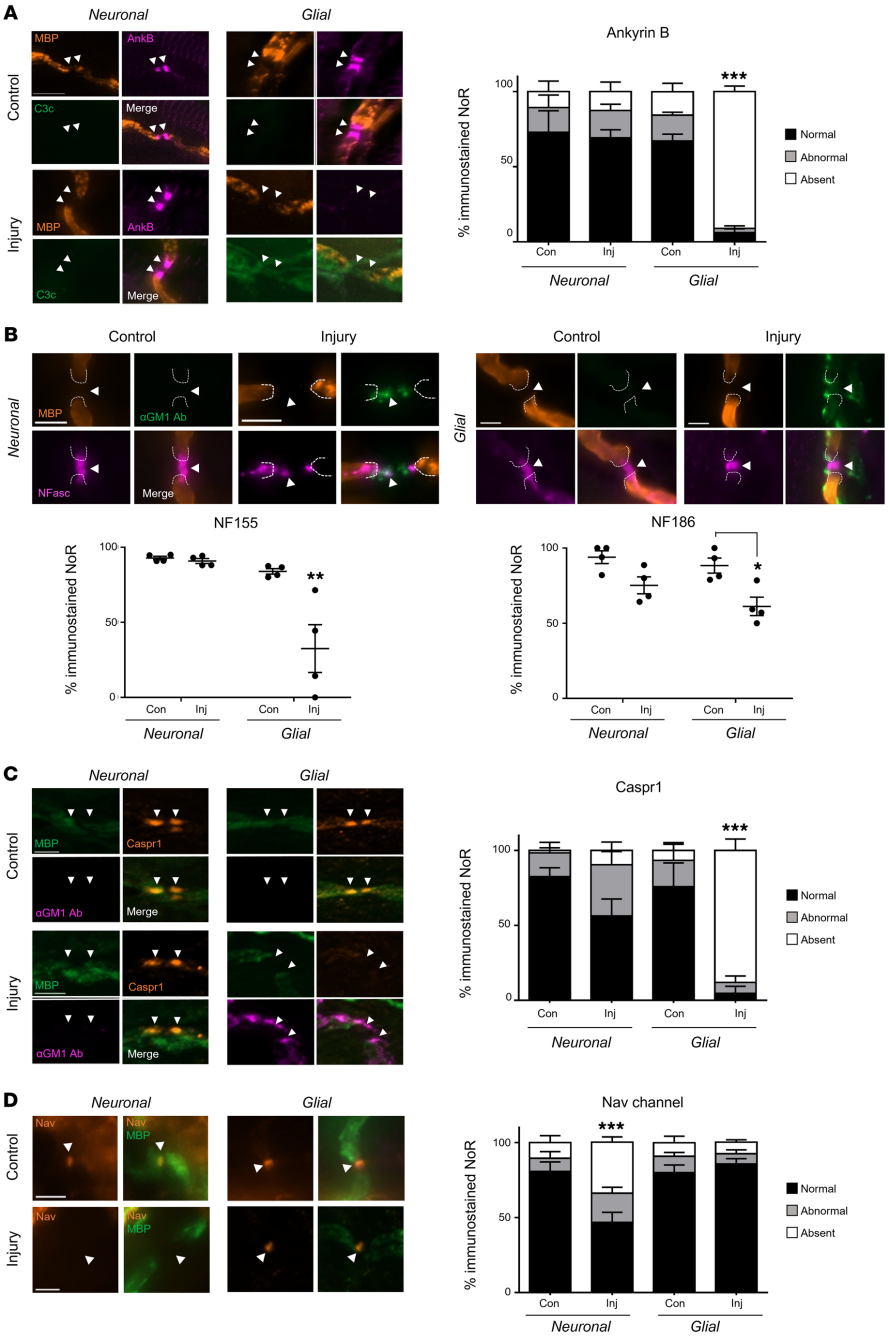
Disruption of paranodal proteins following glial membrane targeting in vivo. *Neuronal* and *Glial* mice were dosed i.p. with 50 mg/kg anti-GM1 Ab followed 16 hours later with 30 μL/g normal human serum (NHS) (injury, Inj) or NHS only (control, Con). The site of expected nodal protein immunostaining is indicated by arrowheads. (**A**) The presence of normal ankyrin B (AnkB) immunostaining at the distal paranode (black bars) was significantly reduced in injured *Glial* mice compared with all treatment groups in the presence of complement (green). (**B**) A pan-neurofascin (Nfasc) Ab was used to assess glial NF155 and axonal NF186 (magenta). Representative images show loss of NF155 staining at paranodal regions, indicated by dashed lines, and the preservation of NF186 when NoRs are decorated with anti-GM1 Ab (green) in *Glial* mice. (**C**) Normal Caspr1 (orange) immunostaining at the distal paranodes was significantly reduced in injured *Glial* mice compared with all other treatment groups. (**D**) There was a reduction in distal NoRs with normal Nav channel (orange) staining in injured *Neuronal* mice. Scale bar: 5 μm. Results are represented as the mean ± SEM. *n =* 4/genotype/treatment: 5–46 NoRs/mouse (median = 21, AnkB); 7–53 NoRs/mouse (median = 25, NFasc); 5–15 NoRs/mouse (median = 11, Caspr1); and 11–27 NoRs/mouse (median = 16, Nav) were analyzed. *P < 0.05, **P < 0.01, ***P < 0.001 for comparisons with the other treatment groups (**A**, **C**, and **D**) or compared with control (**B**) by 2-way ANOVA with Tukey’s post hoc test.

We next investigated injury to the NoRs in vivo following injury. Complement only–treated controls from each genotype showed no significant abnormalities in protein distribution. Anti-GM1 antibody–directed complement-mediated injury to the glial membrane in *Glial* mice resulted in a major reduction in AnkB immunostaining compared with *Neuronal* mice (Figure 5A, *P <* 0.001, two-way ANOVA). NF155 immunostaining was also significantly reduced in injured *Glial* mice compared with all other groups (Figure 5B, *P <* 0.01, two-way ANOVA). NF186-positive NoRs were reduced in both *Glial* and *Neuronal* injured mice in comparison with genotype control mice, but only reached significance in *Glial* mice (Figure 5A, *P <* 0.05, two-way ANOVA, Supplemental Figure 5B).

NoRs with normal Caspr1 immunostaining were significantly decreased in injured *Glial* mice compared with *Neuronal* mice (Figure 5C, *P <* 0.001, two-way ANOVA), where instead there was a greater number of NoRs with disrupted immunostaining; however, this did not reach significance compared to the other groups in vivo. These data indicate that the integrity of the axo-glial junction at the paranode is impaired following direct targeting of glial membranes in injured *Glial* mice in vivo. As observed ex vivo, there was no difference in the intensity of MBP staining along the distal internode among treatment groups (Supplemental Figure 5C), which shows that at this acute time point, injury from glial targeting is restricted to paranodal SC nodal membrane disruption and the myelin sheath remains intact.

We next studied the presence of Nav channel clustering after complement-mediated injury in vivo. Nav channel clustering was unchanged in *Glial* injured mice in this acute injury study; however, injured *Neuronal* mice had significantly fewer NoRs with normal Nav channel staining compared with all other groups (Figure 5D, P *<* 0.001, two-way ANOVA). These results indicate that the localization or presence of axo-glial paranodal proteins are most severely disrupted in *Glial* mice acutely in vivo, while the axonal proteins at the NoRs are only mildly disrupted.

**Figure 6:**
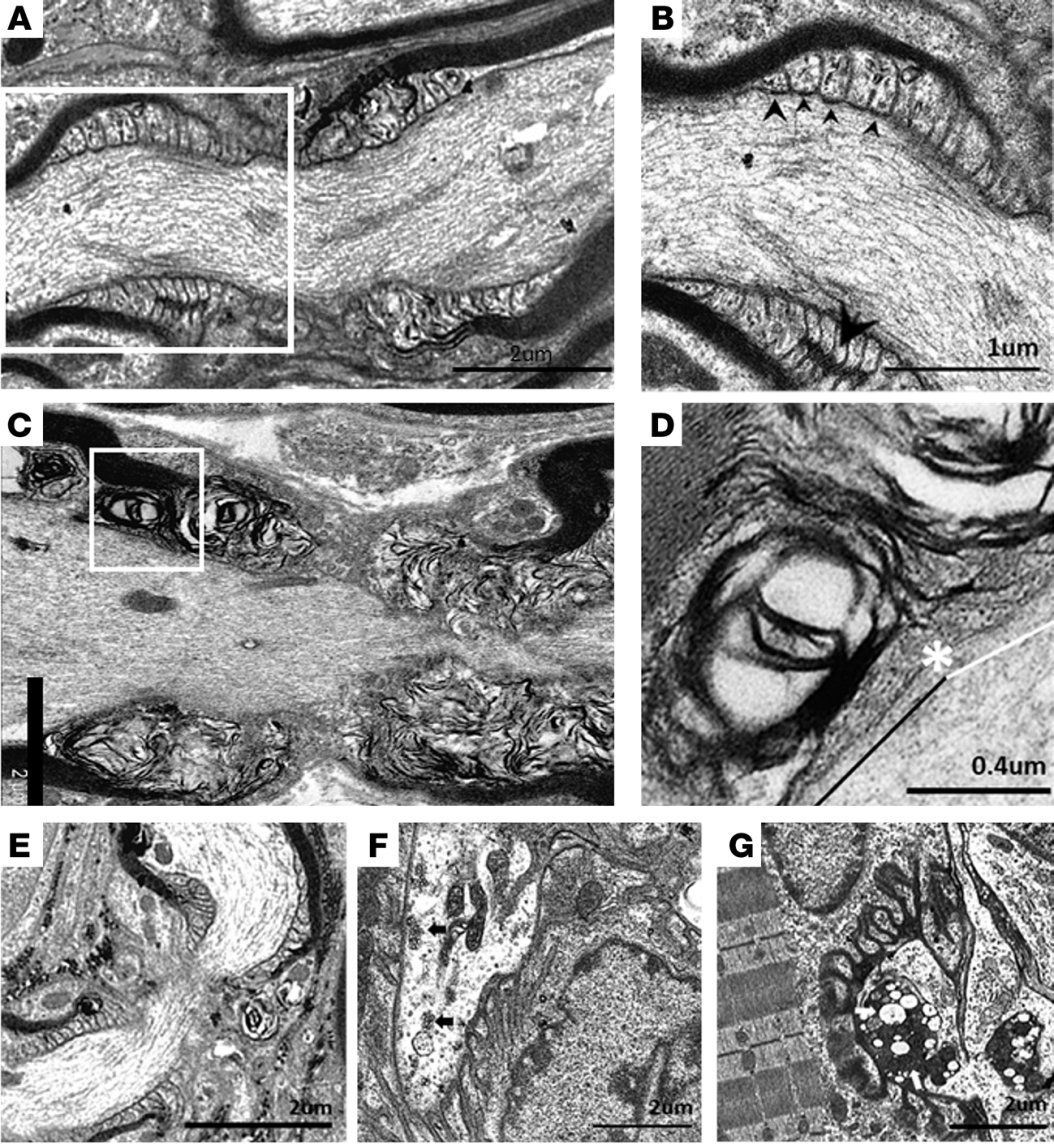
Ultrastructural evaluation of diaphragms from in vivo injury models. *Neuronal* and *Glial* mice were dosed i.p. with 50 mg/kg anti-GM1 Ab followed 16 hours later with 30 μL/g normal human serum (NHS) (injury) or NHS only (control). (**A**) A normal paranode from *Glial* control tissue. N.B. This image is also representative of the *Neuronal* control NoR (not shown). (**B**) Higher magnification of boxed region from **A** shows tight junctions (large arrowhead) between the paranodal loops, and transverse bands (TBs, small arrowheads) at the axo-glial junction between the axon and paranodal loops. (**C**) Injured *Glial* NoRs show severely disrupted paranodal loop organization compared with control. (**D**) Magnification of boxed area from **C**, shows TBs are present between the paranodal loops and axon at the juxtaparanodal-proximal paranode (above black line); however, they are absent at the node-proximal border (above white line, right of asterisk). (**E**) Injured *Neuronal* NoRs show no architectural disruption. (**F**) *Neuronal* control motor nerve terminal (MNT) displays normal architecture and contains synaptic vesicles (black arrows). (**G**) Disturbance to the injured *Neuronal* MNT includes an absence of neurofilament, synaptic vesicles, and the formation of dense or vacuolated mitochondria (white arrows). Results are representative of analysis from 8–10 NoRs per mouse (*n =* 3/genotype/treatment).

In ultrastructural analysis of tissue from in vivo experiments, NoRs from *Glial* control mice showed normal arrangement of paranodal loops forming septate-like junctions with the axon, neurofilaments, SC microvilli, and compact myelin (Figure 6, A and B). In stark contrast, the paranodal loops at NoRs from injured *Glial* mice were highly disrupted, potentially owing to the influx of water and ions through MAC pores (Figure 6, C and D). This arrangement was not observed in any control tissue. SC microvilli remained visible, reflecting gliomedin immunostaining results. At higher magnification, a loss of transverse bands between the node-proximal paranodal loops and axon was observed (Figure 6D, indicated above white line). While NoRs randomly sampled from injured *Neuronal* mice appeared normal (Figure 6E), the architecture of their MNTs showed disruption, with a depletion of neurofilament and synaptic vesicles, and the presence of dense vacuolated mitochondria (Figure 6G) compared with control (Figure 6F), and as seen previously (29). Collectively, these data indicate that in vivo autoimmune injury to the glial membrane can result in paranodal disruption and dysfunction prior to segmental demyelination; this pattern of glial injury differs substantially from the primarily axonal pathology seen in *Neuronal* mice.

### Extended glial injury leads to secondary axonal loss of integrity.

**Figure 7:**
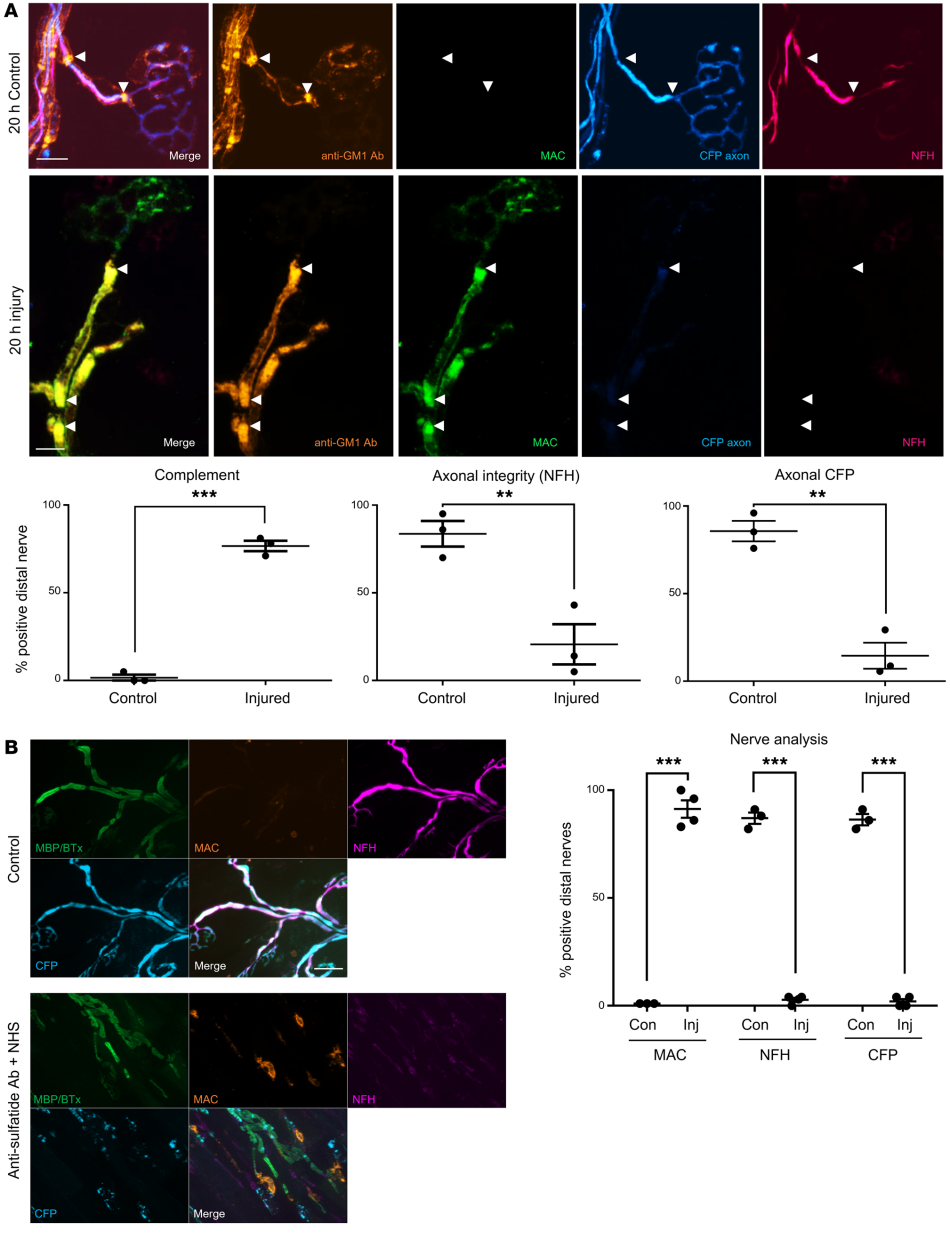
Extended ex vivo injury selectively targeting glial membranes results in secondary axonal degeneration. Triangularis sterni nerve–muscle preparations from *Glial* (**A**) and WT (**B**) mice were treated ex vivo with anti-GM1 Ab or anti-sulfatide Ab, respectively, and a source of complement (injury, Inj) or Ab alone (control, Con) for 20 hours. (**A**) Anti-GM1 Ab (orange) and complement (green) deposition along the distal motor nerve was strongly enriched at the paranodes (arrowheads) in injured compared with control tissue. Loss of axonal integrity along the distal nerve was monitored by presence of neurofilament H immunostaining (NFH, magenta) and cytosolic CFP (blue). (**B**) The experiment was repeated in WT mice using an anti-sulfatide Ab; the results reflect those reported in **A**. Scale bars: 10 μm (**A**) and 20 μm (**B**). Results are represented as the mean ± SEM. *n =* 3/treatment: 25–54 NoRs/mouse (median = 39) were analyzed. **P < 0.01, ***P < 0.001 compared with control by 1-tailed Student’s *t* test.

We next investigated extended injury models both ex and in vivo to look for subacute downstream events, notably loss of axonal integrity that could signify secondary axonal degeneration following selective glial insult (Figure 7A). To this end, only *Glial* mice, including those crossed with axonal cytoplasmic CFP reporter mice, were studied. After 20 hours of anti-GM1 antibody and complement injury treatment ex vivo, antibody and complement deposits were found extensively along the glial membranes, with particular enrichment at the paranodal loops. There was a significant loss in both neurofilament staining and endogenous CFP signal along the entire distal motor nerve of injured tissue. *Glial* control tissue (anti-GM1 antibody only) was positive for antibody staining, negative for complement deposits, and possessed normal intact neurofilament immunostaining and cytoplasmic CFP. To demonstrate that neurofilament loss was secondary to paranodal disturbance and not the product of pSC injury and/or death, we conducted a control ex vivo experiment in WT mice exposed to anti-sulfatide antibody. As pSCs do not form myelin and thus do not express sulfatide, they were not targeted by anti-sulfatide antibody (Supplemental Figure 6), whereas the glial membranes at the paranode, formed by myelinating SCs, were injured (Supplemental Figure 4). As with anti-GM1–treated *Glial* tissue, loss of neurofilament staining and cytoplasmic CFP was also observed in anti-sulfatide antibody–treated mice (Figure 7B), indicating that targeting paranodal glial membranes over 20 hours ex vivo is a sufficient time period to cause subsequent loss of axonal integrity in the distal motor nerve.

**Figure 8:**
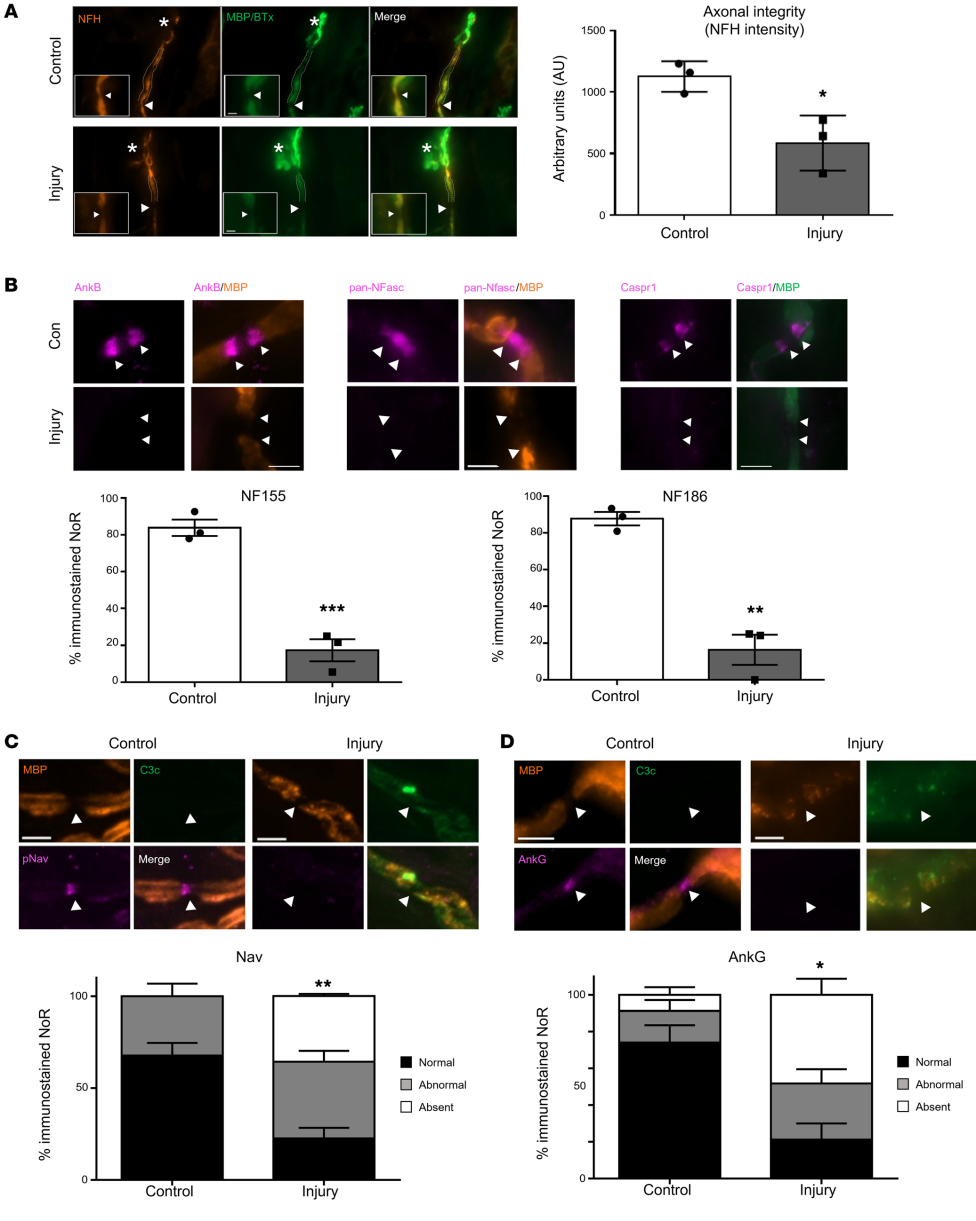
Extended in vivo injury selectively targeting glial membrane results in secondary axonal degeneration. *Glial* mice were dosed i.p. with 50 mg/kg anti-GM1 Ab followed 16 hours later with 30 μL/g normal human serum (NHS) (injury, Inj) or NHS only (control, Con). The experiment was terminated 24 hours after NHS delivery. The site of expected nodal protein immunostaining is indicated by arrowheads. (**A**) At this time point there was loss of neurofilament H staining (NFH, orange) at the motor nerve terminal (MNT, asterisk) and the staining intensity was significantly reduced at the first distal node of Ranvier (NoR). (**B**) Normal ankyrin B (AnkB), NF155, NF186, and Caspr1 (magenta) immunostaining was assessed at distal paranodes after injury compared to control. (**C**) There was a further reduction in distal NoRs with normal voltage-gated sodium (Nav) channel staining (magenta) in injured *Glial* mice compared with control at this extended time point. (**D**) Additionally, the Nav channel–tethering protein AnkG was notably absent. Scale bar: 5 μm. Results are represented as the mean ± SEM. *n =* 3/genotype/treatment: 5–15 NoRs/mouse (median = 11, NFH intensity); 4–25 NoRs/mouse (median = 18, panNFasc); 9–23 NoRs/mouse (median = 12, Nav); and 10–28 NoRs/mouse (median = 18, AnkG) were analyzed. *P < 0.05, **P < 0.01, ***P < 0.001 by 2-tailed Student’s *t* test (**A** and **B**) or 2-way ANOVA with Tukey’s post hoc test (**C**).

Next, we investigated axonal integrity in an extended in vivo injury model in *Glial* mice. At 24 hours after NHS delivery, anti-GM1 antibody and complement deposits were observed along the distal nerve glial membrane in injured diaphragms (data not shown). Unlike the acute injury, neurofilament staining intensity was reduced at the MNT (data not shown) and reached a significant reduction at the first distal NoR in injured mice (Figure 8A, *P <* 0.05, Student’s *t* test).

AnkB, NF155, NF186, and Caspr1 were studied again to determine whether they remain disorganized or recover their normal distribution following initial acute injury. The results demonstrate that AnkB, NF155, and Caspr1 immunostaining remained disrupted 24 hours after injury to the glial membrane (Figure 8B). Moreover, the impaired integrity of the axon at the NoRs progressed over time. There was an enhanced reduction in the number of NF186-positive NoRs in injured *Glial* mice at 24 hours compared with 6 hours (Figure 8B, P *<* 0.01, Student’s *t* test). Additionally, Nav channel and AnkG clustering at the NoRs was significantly reduced in injured mice compared with control, which was not previously significant at the 6-hour time point (Figure 8, C and D, *P <* 0.01 and *P <* 0.05, Student’s *t* test), showing that SC membrane disruption precedes axonal nodal protein disturbances. Overall, these results corroborate the ex vivo findings, confirming that injury to the glial membrane develops over time, and that a loss of paranodal integrity likely precedes and drives secondary bystander axonal loss.

## Discussion

The efforts to distinguish key differentiating features of axonal (AMAN) and demyelinating (AIDP) forms of GBS have developed from widespread clinical observations emerging over the last 30 years, culminating in the recent introduction of the term nodo-paranodopathy to describe forms in which the pathology is centered on the nodal complex (1, 3). The NoR has long been recognized as a site of pathology (6), but the effect, if any, of injury to this site on bystander or secondary axonal degeneration is little understood. Herein, we have developed a model system to investigate this injury that allows us to mechanistically separate axonal from SC membrane dysfunction unambiguously, using a single monoclonal autoantibody against the prototypic GBS nodal antigen, GM1 ganglioside.

The segregation of GM1 to axonal or glial membranes (20) permits us to study selective injury to either membrane at the axo-glial interface, and thereby to distinguish mechanisms underlying primary and secondary axonal failure and degeneration. In ex vivo preparations this was compared to the outcomes in WT preparations where both neural membranes are targeted. While it would be ideal to model anti-GM1 antibody–mediated neuropathy in vivo in WT mice, the major handicap has been WT mouse resistance to developing GBS following passive immunization. We have attributed this to the low levels of complement activity in mice, the sequestration and depletion of circulating anti-GM1 antibody by non-neural tissues, and protection afforded by the blood-nerve barrier (16, 28, 30). The model described herein removes the non-neural expression of GM1, thereby raising circulating antibody to pathogenic levels, uses NHS as a potent source of heterologous complement, and studies distal motor nerves projecting out with the blood-nerve barrier. The combined application of these 3 steps allows us to observe striking clinical and morphological phenotypes in vivo. Topical application of reagents in our ex vivo paradigm reflect a severe disease pathology. Owing to the injury at the MNT resulting in synaptic dysfunction with severe respiratory distress, the in vivo experiment was restricted to 6 hours for *Neuronal* mice, meaning that this model represents a milder stage of injury and explains the differences in injury reported between ex and in vivo. In the future, a subacute injury paradigm with a milder clinical phenotype (31) could be applied to *Neuronal* mice to study nodal changes in vivo in more detail.

### Delayed loss of axonal integrity following glial membrane injury.

Our results show that anti-GM1 antibody binding can be selectively targeted to, and activate complement on, axonal or glial membranes at distal NoRs and MNTs. Loss of neurofilament immunostaining, indicating a loss of axonal integrity, follows acutely in *Neuronal* but not *Glial* tissue, suggesting direct targeting of the axonal membrane, as previously observed in other models of AMAN (16, 21). However, most significantly, we find that following early paranodal disruption and nodal axonal protein disturbances in *Glial* mice, axonal integrity is compromised following extended glial injury, thus indicating that indirect axonopathic mechanisms must account for subsequent axonal loss in the extended model. These results, first seen ex vivo, were then recapitulated in vivo in an extended *Glial* injury model. Notably, our results also confirm that despite pSC injury in our *Glial* paradigm, acute injury to these specialized glial cells does not account for the acute loss of axonal integrity seen in the distal motor nerve, aligning with previous observations (15, 32). A diverse array of mechanisms have been proposed to contribute to secondary bystander axonal degeneration including, but not limited to, demyelination, mitochondrial dysfunction, and disrupted trophic support (33). Because of the proximity of axonal and glial membranes in the nodal complex, it is possible that cytotoxic mediators are released from the injured glial membrane that could indirectly cause axonal damage. There is no evidence that MAC pores can be shed and reinserted intact into another adjacent membrane (34); however, MAC formation is a highly toxic injury resulting in a wide range of uncontrolled bidirectional solute fluxes. Based on our data, herein we will discuss the contribution of paranodal axo-glial and nodal protein disturbances to axonal degeneration.

### Selective membrane targeting leads to loss of axo-glial integrity and function through different pathways.

To account for the acute cascade of molecular events leading to collapse of the axo-glial interface at the paranodal junction, our results suggest a differential mislocalization of proteins dependent on 4 factors: (a) local anchoring of CAMs through interactions with cytoskeletal adaptor proteins; (b) axo-glial stabilization and tethering through axo-glial *trans* interactions; (c) calpain substrate status of intracellular cytoskeletal adaptor protein domains; and (d) distortion of the paranodal loops and loss of integrity. In this study we interpret a loss of immunostaining as the absence or dispersion of a protein. For the abundant cytoskeletal protein neurofilament, absence is an indicator of compromised axonal integrity, which could progress to axonal loss/degeneration. Loss or disruption of nodal protein immunostaining may be through direct calpain cleavage of known substrates (AnkB, AnkG, and Nav) (35, 36), or indirectly through cleavage of known tethering proteins causing dispersion, detailed below. Injury to *Neuronal* tissue reflects findings previously observed in AMAN models and is likely driven by MAC pore formation, with intra-axonal calcium influx and calpain activation (14, 16). This likely leads to the following sequence of events: calpain cleavage of axonal cytoskeletal adaptor protein AnkG; dispersion of NF186; SC microvilli gliomedin remains present; Caspr1 loss at the node-proximal paranode; consequent perturbation of the Caspr1 binding partner NF155, and glial AnkB; Nav channel cleavage and/or cluster dispersion; and conduction failure. Conversely, *Glial* injury to NoRs likely leads to the following sequence of events: MAC-induced paranodal loop distortion; calpain cleavage of glial paranodal scaffold protein, AnkB; disruption of paranodal NF155 through general loop distortion and loss of partial interaction with AnkB; consequent disturbance of axonal Caspr1 binding partner; mislocalization and partial loss of gliomedin; dispersion of axonal NF186; followed by Nav channel cluster and AnkG dispersion and conduction failure. Despite these differences, the effect on Nav channel clustering and function is the same, likely owing to the loss of 2 Nav channel clustering mechanisms: cytoskeletal anchors and an intact paranode.

Inducible ablation has shown that NF186 has a critical role in maintaining PNS Nav clusters at the NoR (37), and NF186 stability can underlie long-term Nav channel clustering and axon health in concert with the paranodes (38). In *Neuronal* mice, AnkG is likely directly cleaved, and Caspr1 is indirectly disturbed through calpain cleavage of the axonal cytoskeletal proteins 4.1B and βII spectrin that tether it to the cytoskeleton (39). These axonal paranodal cytoskeletal proteins are critical in combination with NF186 for Nav channel positioning; combined paranodal βII spectrin and NF186 conditional deficiency causes impaired AnkG and Nav channel clustering (40), reflecting our observations. Combined with Nav channel clustering loss in our *Neuronal* model, the presence of MAC pores in the axonal membrane drives ionic imbalance, culminating in loss of membrane potential and function, as previously observed (16). These disturbances culminate in conduction block and nodal lengthening likely through paranodal disruption from loss of node-proximal Caspr1, or membrane insertion as observed in patient autopsy tissue (6).

When the glial membranes are selectively targeted, we propose the loss of the axo-glial junction proteins at the node-proximal paranodal loops begins with distortion, swelling, and loss of paranodal loop integrity through the influx of water and ions via MAC pores, as observed in ultrastructural images. Loss of the calpain substrate AnkB immunostaining is an indicator of MAC-pore-induced paranodal loop disruption. While AnkB loss is not entirely causal, it could contribute to the disruption of NF155 through their partial interaction. Normally, NF186 maintains Nav channel clusters through interactions with SC microvilli extracellular matrix proteins, including gliomedin (17). Therefore, we propose that the combined disturbance of SC gliomedin and the axo-glial CAMs leads to Nav channel dispersion, which underlies, in part, the ensuing conduction failure. Chang et al. (24) found no change to NF155 and Caspr1 following conditional ablation of AnkB; however, they propose a compensation by substitution with other glial cytoskeletal spectrins and actins. These proteins would presumably also be cleaved in our acute injury setting, thereby preventing this substitution from becoming functional and exacerbating the disruption. It is true that mice deficient in axo-glial CAMs have minimal disturbances in Nav channel clustering (41–43), likely due to the presence of NF186. But here, similar to *Neuronal* injury, a disturbance of both NF186 and paranodal integrity causes Nav channel cluster loss in *Glial* mice. Additionally, paranodal loop disruption also likely leads to a leakage of driving current due to disruption of axo-glial junctional scaffolds causing conduction block, as previously described (3).

Conduction failure can be transitory and reversible, or irreversible if the axons ultimately undergo transection with subsequent Wallerian degeneration. The tipping point — a concept also referred to as the metastable state — that dictates the capability of injured axons to recover through local repair in favor of complete axonal transection is likely to be mechanistically multifactorial (44). Therefore, distinguishing the outcome of immune attack on axonal or glial membranes that have similar electrophysiological outcomes has clear implications for therapeutic strategies and recovery. Whether, over time, our current models would proceed to segmental demyelination, either directly, or as a result of axonal transection with Wallerian degeneration, or be restricted to focal membrane injury around the nodal region, remains to be determined. However, from existing evidence it is reasonable to predict that both segmental demyelination and/or Wallerian degeneration would be demonstrated in a prolonged-injury model.

### Loss of axo-glial integrity and secondary bystander-axon degeneration.

It has been shown that the axo-glial CAMs NF155, Caspr1, and contactin-1 assemble a specialized axonal cytoskeleton at the paranode (protein 4.1B, αII and βII spectrin), which promotes nodal domain formation and anchoring to the actin cytoskeleton (17). Additionally, it has been proposed that axonal actins and spectrins form a periodic cytoskeleton with submembranous rings, and coalignment of submembranous paranodal cytoskeletal proteins is critical to arranging the paranodal CAMs (17). Our Kv1.1 data from the acute injury paradigm suggests the localization of these cytoskeletal proteins remains intact, as there is no invasion into the paranodal domain. However, the delayed consequence of a distortion to this regimented arrangement by a disturbance in glial CAM localization could be secondary loss of axonal integrity, manifest as a porous membrane. In a recent model of experimental autoimmune encephalomyelitis, calcium reporter mice were used to monitor calcium entry into axons traveling through inflammatory lesions (45). The authors discovered that extracellular calcium ions were entering through nanoruptures and levels could ultimately predict axonal fate. They predict these nanoruptures could be caused by toxic mediators or mechanical forces, the latter neatly aligning with the proposed disturbances to the regular pattern of the actin/spectrin axonal cytoskeleton in our extended model. Cytoplasmic CFP loss in our extended model strongly indicates a loss of axonal membrane integrity. Furthermore, neurofilament is a known calpain substrate and its loss in the same paradigm points toward calcium entry and calpain activation as the final executioner.

### Clinical significance.

A recent clinical development has been the introduction of the term nodo-paranodopathy as an explanatory concept focused on this site that in part escapes from the binary AMAN/AIDP classification (3). In parallel with these advances in clinical and electrophysiological observations, little effort has been applied to developing animal models that might support them experimentally. Exploring the mechanisms underlying the nodo-paranodopathy concept will undoubtedly benefit from the generation of the *Neuronal* and *Glial* transgenic mice and other relevant models (46).

The pathological situation of acute anti-GM1 antibody–associated GBS in humans, be that AMAN, AIDP, nodo-paranodopathy, or a hybrid form, would likely resemble the situation described herein in the WT mouse. There are, however, many unknown factors that make this assumption of human-murine parity overly simplistic. Firstly, the distribution and concentration of GM1 in human axonal and glial membranes relative to the mouse is unknown. Secondly, both human and mouse polyclonal and monoclonal anti-GM1 antibodies differ considerably in their ability to bind to GM1 in different membrane environments. *Cis* interactions between GM1 and other molecules in the plane of the plasma membrane shape the topographical presentation of GM1 epitopes for antibody binding that may be enhancing or inhibitory (47). Thus, certain anti-GM1 antibodies may preferentially favor binding to glial GM1 versus axonal GM1, or vice versa. In the context of anti-GM1 antibody–associated GBS this could affect the AMAN/AIDP relative balance. Notwithstanding these considerations, it seems likely that anti-GM1 antibody–associated GBS in humans would be driven by a summation of direct and indirect axo-glial injury mechanisms that may evolve at different rates over time according to antibody titer, specificity, and host-intrinsic inflammatory and axonal vulnerability factors.

The specialized nature of the distal nerve site studied over a relatively brief time period is a constraint to translating our current model into a human homolog, although this distal site is certainly affected in human GBS (2, 3). Looking forward, further extending this model over time in vivo, subject to animal welfare constraints, will allow us to elucidate the mechanism of secondary bystander axonal degeneration, or reversible conduction failure, and thereby add mechanistic insights into the likely effects of novel treatments in humans. Characterizing SC membrane and myelin injury will allow us to study whether these early disease stage findings exemplify reversible axonal damage or can lead to more permanent axonal degeneration; shifting the metastable state in favor of local repair rather than axonal transection (44) could have major effects on clinical outcome. Indeed, we observed some mild changes to myelin (vesiculation) and EthD-2–positive internodal nuclei that show signs of early SC body injury, the likely forerunner of segmental demyelination. SC nodal membrane disruption is just one of many mechanisms that occur in response to anti-GM1 antibody–mediated injury and comprise the path to degeneration. While certainly affected in human GBS, the distal nerve is a site with specialized features that must be taken into consideration. Distal axons are small in diameter and vulnerable to damage, and the pSCs are capable of promoting rapid repair and regeneration after axonal injury (31). Exploring these factors in models remains highly relevant to understanding the highly complex axo-glial interplay at the different anatomical sites affected in human GBS.

## Methods

### Antibodies and reagents

The majority of experiments used the mouse monoclonal IgG3 anti–GM1 ganglioside antibody, DG2. This was generated by immunizing ganglioside-deficient mice with ganglioside liposomes or ganglioside-mimicking *Campylobacter jejuni* LOS, as described previously (22). Mouse monoclonal IgG3 anti-sulfatide antibody was also used, and generated as previously described (27). The following antibodies were used for immunostaining studies to identify proteins and complement: mouse anti-AnkB (UC Davis/NIH NeuroMab Facility, N105/17; 1:300), mouse anti-AnkG (Thermo Fisher Scientific, 33-8800; 1:100); rabbit anti-Caspr1 (gifted by Elior Peles, Weizmann Institute, Rehovot, Israel; 1:1000); FITC-labeled rabbit anti-C3c (Agilent, Q036805; 1:300); rabbit anti-gliomedin (Abcam, ab24483; 1:100); mouse anti–human C5b-9 (Agilent, M0777; 1:50); rabbit anti-Kv1.1 (Alomone Labs, APC-009; 1:200); rat anti-MBP (Bio-Rad, MCA409S; 1:500); mouse anti–pan Nav (pNav; Sigma-Aldrich, 8809; 1:100); rabbit anti-Nav1.6 (Sigma-Aldrich, S0438; 1:100); rabbit anti–pan neurofascin (anti-panNFasc; gifted from Peter Brophy, University of Edinburgh, Edinburgh, UK; 1:1000); and mouse anti–phosphorylated neurofilament-H antibody (NFH, BioLegend, 801602, clone SMI31; 1:1500). Secondary antibodies were as follows: isotype-specific Alexa Fluor 488– and Alexa Fluor 647–conjugated goat anti–mouse IgG3 (Thermo Fisher Scientific, A-21151; 1:500), IgG2a (Thermo Fisher Scientific, A-21131; 1:500), and IgG1 antibodies (Thermo Fisher Scientific, A-21240; 1:500); Alexa Fluor 488– and Alexa Fluor 555–conjugated goat anti–rabbit IgG (Thermo Fisher Scientific, A-21429 or A21446; 1:500); and goat anti–rat IgG antibodies (Thermo Fisher Scientific, A-21434; 1:500). To identify the postsynaptic membrane, fluorescently labeled α-bungarotoxin was used (Molecular Probes, T1175; 1:500). To assess cell viability, the nucleic acid stain EthD-2 (Thermo Fisher Scientific/Invitrogen, E3599; 1:500) was used.

For ex vivo injury preparations, tissue was maintained alive in oxygenated (95% O_2_/5% CO_2_) Ringer’s solution (116 mM NaCl, 4.5 mM KCl, 1 mM MgCl_2_, 2 mM CaCl_2_, 1 mM NaH_2_PO_4_, 23 mM NaHCO_3_, 11 mM glucose, pH 7.4). Ex vivo electrophysiological preparations were maintained alive in oxygenated Tyrode’s solution (137 mM NaCl, 2.7 mM KCl, 0.5 mM MgCl_2_, 2.5 mM CaCl_2_, 0.4 mM KH_2_PO_4_, 11.9 mM NaHCO_3_, 10 mM HEPES, 11 mM glucose, pH 7.4). For ex vivo preparations, all primary antibodies were applied in a blocking solution (3% normal goat serum [NGS] + 0.5% Triton X-100 in PBS). For diaphragm sections from in vivo experiments, antibodies were prepared in PBS plus 3% NGS and 0.1% Triton X-100. Secondary antibodies were diluted in PBS plus 1% NGS. NHS was collected from a single donor, rapidly frozen, and stored in multiple aliquots at –70°C to preserve complement activity.

### Mice

WT mice (Harlan) and 2 previously described transgenic strains, *GalNAc-T^–/–^-Tg(neuronal)* (21) and *GalNAc-T^–/–^-Tg(glial)* (20), all on a C57BL/6 253 background backcrossed for 7 generations, were used. Briefly, *GalNAc-T^–/–^-Tg(neuronal)* mice express the full-length cDNA encoding GalNAc-T under the control of the human *Thy1.2* promoter (restricted to mature neurons), and *GalNAc-T^–/–^-Tg(glial)* mice express the full-length cDNA encoding GalNAc-T under the control of the mouse *Plp* promoter (restricted to glia, including myelinating and nonmyelinating SCs). Additionally, mice were crossed with B6.Cg-Tg mice that endogenously express cytosolic cyan fluorescent protein (CFP) in their axons (21). Mice from the *GalNAc-T^–/–^-Tg(neuronal)* or *GalNAc-T^–/–^-Tg(glial)* background exhibit age-dependent neurodegeneration (20); therefore, we elected to use 4- to 6-week-old mice, both male and female, that have no identifiable phenotype (12). The number of mice per treatment are reported per experiment. Mice were maintained under a 12-hour light/dark cycle in controlled temperature and humidity with ad libitum access to food and water. For each study, mice were euthanized by increasing-CO_2_ inhalation.

### Ex vivo injury models

#### Nerve integrity.

Ex vivo TS nerve–muscle preparations from WT, *Neuronal*, and *Glial* mice were used for each study as described previously with some modifications (21). Each mouse provides 2 TS: one half was used as an uninjured control (anti-GM1 antibody only) and the second half as the injured preparation (anti-GM1 antibody plus NHS). For the acute injury model, all TS were incubated for 4 hours at 32°C in Ringer’s with 100 μg/mL anti-GM1 antibody and additionally 40% NHS for the injury half. For the extended injury model, TS were incubated for 20 hours at room temperature. The TS were washed 3 times in Ringer’s, followed by a 1-hour incubation with anti-MAC antibody at 4°C. For the EhtD-2 homodimer live stain (2 mM), the label was applied for 30 minutes at room temperature prior to fixation. The tissue was washed 3 times in Ringer’s followed by fixation with 4% paraformaldehyde (PFA) at 4°C. Washes with PBS, 0.1 M glycine, and PBS followed. TS were transferred to 100% ethanol for 10 minutes at –20°C, and then thoroughly washed in PBS. Tissue was incubated overnight at 4°C in blocking solution plus mouse anti-MAC antibody, rat-anti MBP antibody, and α-bungarotoxin with one of the neural markers under investigation. TS were rinsed 3 times in PBS followed by a 2-hour incubation with secondary antibodies at room temperature in the dark. TS received final washes in PBS and were mounted in Citifluor mounting medium (Citifluor Products). For each neural marker, *n =* 3/4 mice per genotype.

#### Perineural recordings.

Perineural recordings were made from TS nerve–muscle preparations set up as described previously (16, 48), with some modifications. Briefly, preparations were set up for electrophysiological recordings in Tyrode’s solution following a 3-hour incubation of the TS with 100 μg/mL anti-GM1 antibody plus 40% NHS in Tyrode’s at 32°C. Antibody only and NHS only controls were used. For each of the 3 treatments, *n =* 4 mice/genotype.

### In vivo injury model

The in vivo model used here is based on the axonal injury model previously described (21) with a few modifications. This is a nonrecovery model for *Neuronal* mice and due to its severe clinical phenotype, the time course was restricted to 6 hours for this genotype. Briefly, WT, *Neuronal*, and *Glial* mice were i.p. injected with 50 mg/kg anti-GM1 monoclonal antibody, or the equivalent volume of PBS for control groups. NHS (30 μL/g) was delivered i.p. 16 hours later. Whole-body plethysmography (EMMS) recordings were then collected from 3.5 to 5 hours. Flow-derived parameters of breath frequency and tidal volume were collected from 25 accepted breaths and averaged over 25 readouts at 5 hours after injury. All recordings were performed using eDacq software (version 1.9.4, EMMS). Mice were asphyxiated with an increasing concentration of CO_2_, and terminal blood samples were taken for ELISA and complement assay. The diaphragm was collected for immunohistological or ultrastructural analysis: half was snap frozen immediately, the other half was fixed in 4% PFA for 1 hour at 4°C, and then washed in PBS. For each treatment, *n =* 4 mice/ genotype. The extended in vivo injury model was performed as described above except mice were culled at 24 hours after NHS injection (*n =* 3 mice per treatment).

#### In vivo immunofluorescence.

Diaphragm sections (10 and 15 μm) were collected onto 3-aminopropyltriethoxysilane–coated slides and used for complement/nodal and neurofilament analysis, respectively, at the MNTs and NoRs. Three slides were prepared for each label and first blocked for 1 hour at 4°C prior to antibody incubation overnight at 4°C. Slides were washed in PBS, followed by incubation with secondary antibodies at room temperature for 2 hours in the dark. Slides were again washed in PBS and mounted in Citifluor.

#### Serum ELISA.

ELISAs were performed to confirm the presence of anti-GM1 monoclonal antibody in the mouse sera as described previously (21).

#### Ultrastructure.

After the completion of in vivo experiments (*n =* 3/treatment), the diaphragm was collected and either immersion fixed or transcardially perfused with a 5% glutaraldehyde/4% paraformaldehyde mixture before being processed for resin embedding as previously described (49). Grids were cut for ultrastructural analysis and illustrative electron micrographs of distal nerves captured on a Jeol 1200 EX transmission electron microscope with Cantega 2K × 2K digital camera.

### Image acquisition and quantification

All imaging was performed using a Zeiss Z1 Imager with ApoTome attachment, or Zeiss LSM 880 confocal microscope and captured with Zen software (ZEN Digital Imaging for Light Microscopy). A 40× or 63× oil objective was used to capture snaps or *Z*-stacks (0.4 μm interval). From these images, 18 to 133 MNTs and 5 to 53 NoRs/treatment/mouse were analyzed. Distal MNTs were identified using α-bungarotoxin and distal NoRs were identified by a gap in MBP immunostaining. Illustrative images are maximum intensity projections of captured z-stacks. Nerve terminal occupancy was classified as follows: occupied if complement or neurofilament staining overlaid α-bungarotoxin, otherwise categorized as absent/unoccupied. Loss of neurofilament staining was considered a loss of axonal integrity, which could ultimately signify axonal loss. Scoring for presence and normality of nodal protein (AnkG, NF186, Nav1.6, gliomedin) or paranodal protein immunostaining (AnkB, NF155, Caspr1) was as follows: NoRs were classified into 3 groups: (a) Normal = present and normal; (b) Abnormal = present but abnormal, i.e., fragmented cluster/widened nodal gap/weakened staining; (c) Absent. If only 1 paranodal protein domain remained then this was categorized as absent. FIJI software (Fiji) was used to quantify intensity of neurofascin and MBP across the NoRs. Fluorescence intensity centered on the nodal gap was plotted for each marker. For all ex vivo analysis of the effect of complement on neural structures, only NoRs positive for complement deposition in the injured group were included for quantification.

### Experimental design

For in vivo experiments, mice were randomly allocated to treatment groups using a random number generator. Animals were excluded from the injury treatment group if the serum was absent for AGAb or complement assessed by ELISA and topical complement assay, respectively. A power analysis was performed using G*Power software v3.0.10 to determine group size; *n =* 3 (ex vivo) and *n =* 4 (in vivo) was selected for each treatment group. Effect size for behavioral output was based on previous experiments, calculations made on the basis of 80% power, and 0.05 significance criteria. All tissue was coded to prevent researcher bias.

### Statistics

The number of independent animals are described in the Methods and indicated in the figure legends. Statistical differences among genotypes were determined by Student’s *t* test, 2-way ANOVA for multiple factors, or repeated measures 2-way ANOVA followed by a Fisher’s, Tukey’s, or Bonferonni’s post hoc test for multiple comparisons using GraphPad Prism 7 software. Parametric testing was used, and differences were considered significant when *P* values were less than 0.05. Data were plotted as the mean ± SEM using dot plots or contingency graphs.

### Study approval

All experiments using mice conformed to University of Glasgow institutional guidelines and complied with relevant guidelines on the care and use of animals outlined in the revised Animals (Scientific Procedures) and were performed in accordance with a license approved and granted by the United Kingdom Home Office (POC6B3485).

## Author contributions

RM and HJW designed the research and wrote the manuscript. RM, CIC, JAB, MEC, DY, and EGR conducted experiments and acquired and analyzed the data. CLC and SR performed pilot studies. RM, CIC, JAB, MEC, CLC, SR, EGR, and HJW reviewed the manuscript.

## Supplementary Material

Supplemental data

## Version 1 06/07/2022

In-Press Preview

## Version 2 07/15/2022

Electronic publication
