# Supplementary material for: Schwann cell nodal membrane disruption triggers bystander axonal degeneration in a Guillain-Barré syndrome mouse model
Source: J Clin Invest. 2022 Jul 15;132(14):e158524. doi: 10.1172/JCI158524 (PMC9282931; doi:10.1172/JCI158524)
Supplement: Supplemental data [file jci-132-158524-s102.pdf]

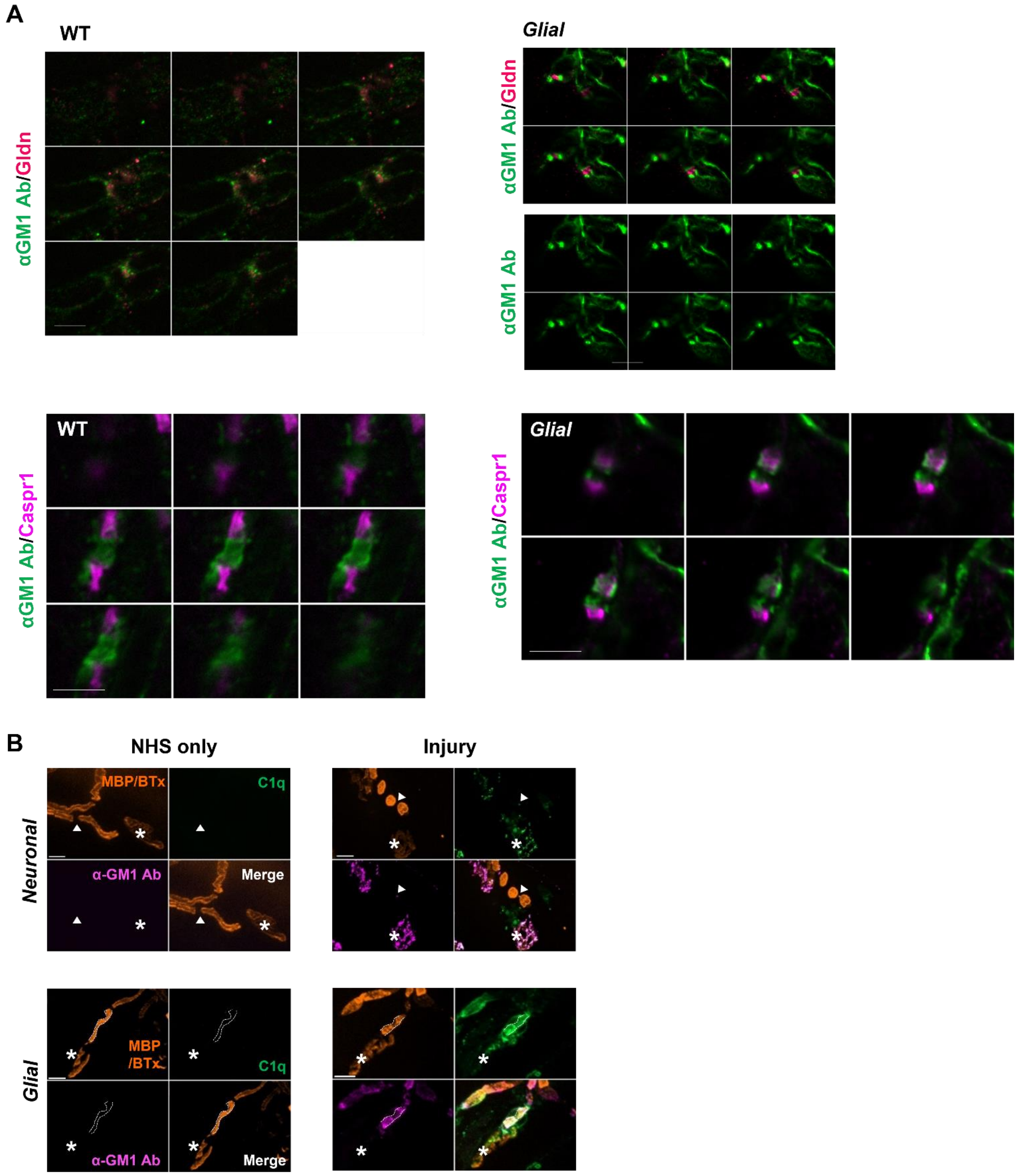

Supplementary Figure 1. Anti-GM1 Ab and complement localisation in distal nerve. A) Triangularis sterni (TS) nerve-muscle preparations from wild type (WT) and *Glial* mice were treated ex vivo with anti-GM1 Ab alone (green). Single optical images from a z-stack taken through nodes of Ranvier (NoR) co-labelled with either gliomedin (orange) or Caspr1 (magenta) show clear nodal axolemmal anti-GM1 Ab staining at WT NoR but not detectable in *Glial* mice. B) TS preparations from *Neuronal* and *Glial* mice were treated ex vivo with anti-GM1 Ab and a source of complement (injury, Inj) or normal human serum (NHS) only for 4h at 32°C. Nerves are identified by labelling with myelin basic protein (MBP, orange) and bungarotoxin (BTx, orange, asterisk). Ab (magenta) and complement (C1q, green) were absent in NHS only control from both genotypes. After injury Ab and complement were present on the axon terminal (asterisks) and nodal axolemma (arrowheads) in *Neuronal* mice, while Ab and complement is along the SC membrane (dashed lines) in *Glial* mice. Scale bar = 5µm (A) 10µm (B).

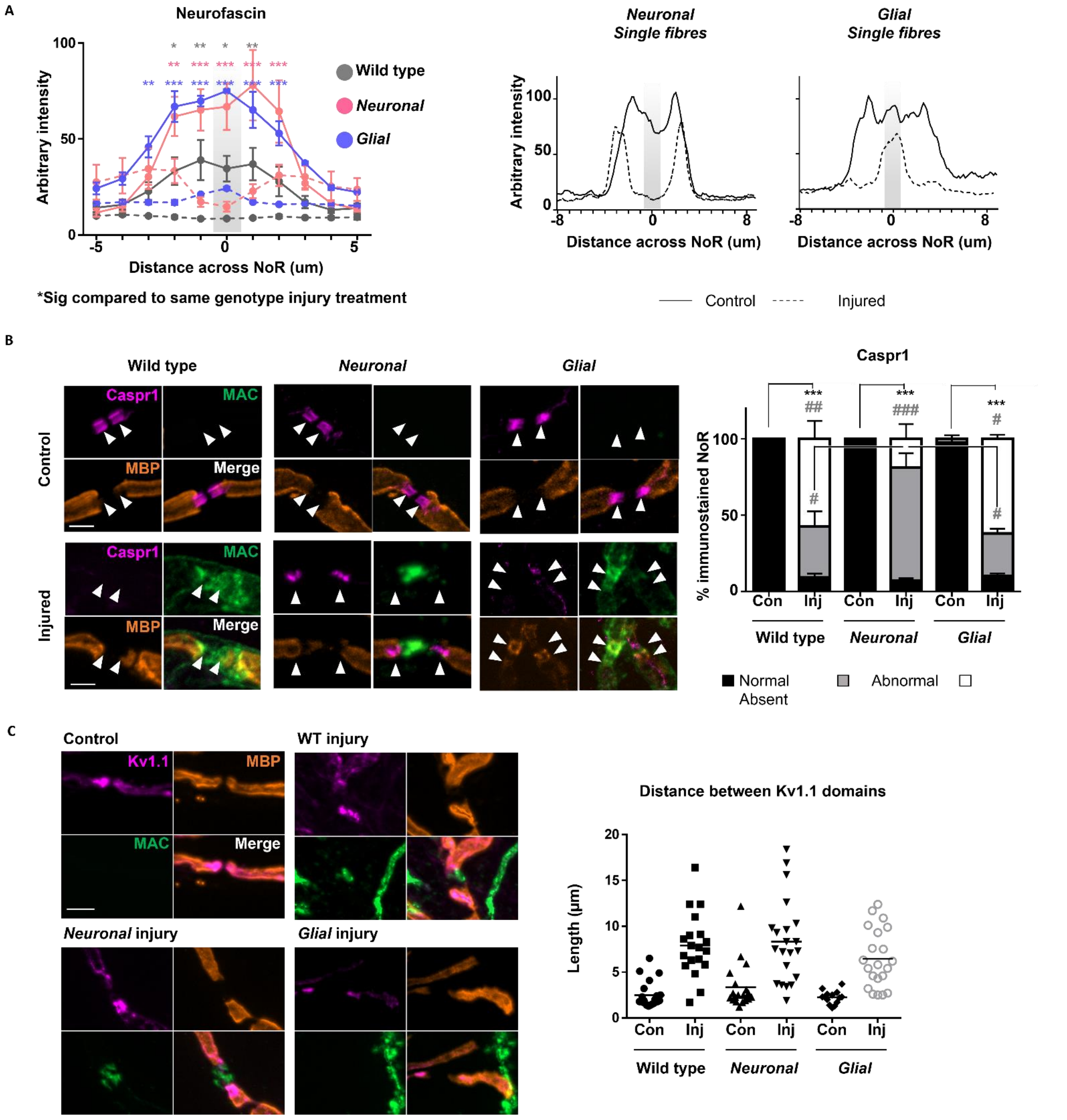

Supplementary Figure 2. Neurofascin, Caspr1 and Kv1.1 channel integrity following neuronal and glial membrane injury ex vivo. Triangularis sterni nerve-muscle preparations from wild type (WT), *Neuronal* and *Glial* mice were treated ex vivo with anti-GM1 Ab and a source of complement (injury, Inj) or anti-GM1 Ab alone (control, Con) for 4h at 32°C. NoR were identified by a gap in myelin basic protein (MBP, orange) immunostaining. A) The intensity of panNfasc immunostaining across the node centred on the nodal gap (grey shading) for each genotype is shown. Results show that all control tissue had a wide uniform trace, extending across the NoR. Representative examples of individual nodal profiles from *Glial* and *Neuronal* tissue are shown. B) In WT and *Glial* injured tissue there was a significant loss (*, white bars) or abnormality (#, grey bars) of Caspr1 immunostaining at NoR compared to controls. C) In a preliminary study (n=1/treatment) the gap between Kv1.1 channel domains lengthened under injury condition in all genotypes and demonstrated no clear paranodal invasion. Results are represented as the mean ± SEM. n=3/genotype/treatment; 10-20 NoR/mouse were analysed (pNFasc,MBP), 12-30 NoR/mouse (median=19, Caspr1) were analysed. Two-way ANOVA, * signifies p<0.05, ** signifies p<0.01 and *** signifies p<0.001. n=1/genotype/treatment; 15-23 NoR/mouse (Kv1.1) were analysed. Scale bar = 5µm.

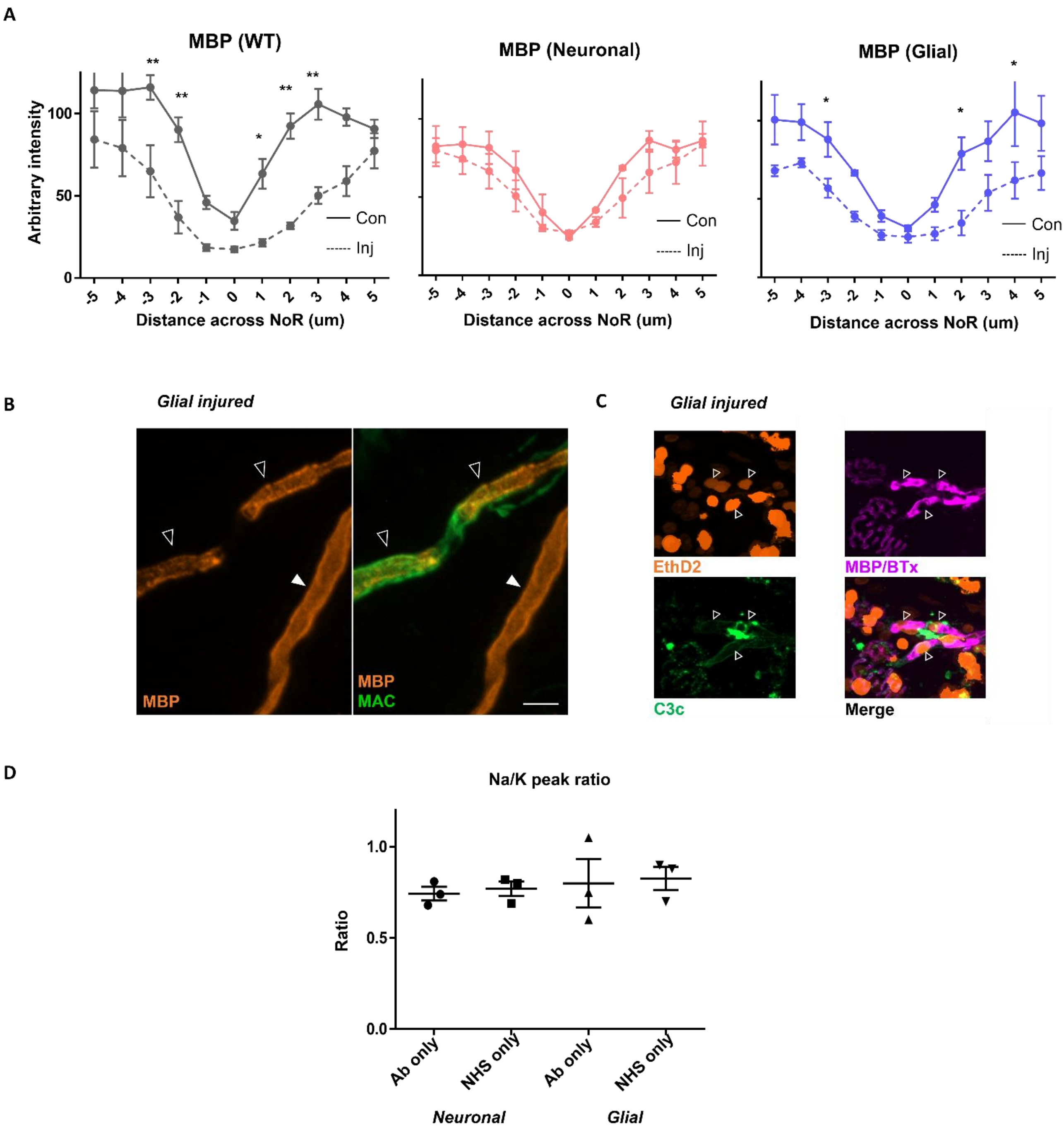

Supplementary Figure 3. Myelin integrity following neuronal or glial membrane injury ex vivo. Triangularis sterni nerve-muscle preparations from wild type (WT), *Neuronal* and *Glial* mice were treated ex vivo with anti-GM1 Ab and a source of complement (injury, Inj) or anti-GM1 Ab alone (control, Con) for 4h at 32°C. NoR were identified by a gap in myelin basic protein (MBP, orange) immunostaining. A) In control mice the MBP intensity is greatest at the paranodes and dips in the centre where the nodal gap lies and MBP is absent. The intensity at the node-proximal paranodes falls in *Glial* and WT injured NoR and there was no change to MBP staining at *Neuronal* NoR. B) Intensity of MBP along the internode does not change in injured groups, but there is an abnormal appearance. An example of normal MBP staining (orange, closed arrowheads) and abnormal punctate-like MBP staining (open arrowheads) when co-localised with complement deposition (green) in *Glial* injury tissue is shown. Scale bar = 5 um. C) In addition to abnormal myelin appearance, Schwann cell nuclei were positive for EthD2 (orange) when decorated with complement (green) in *Glial* injured tissue, indicating early damage to this cell. Distal nerves were identified by bungarotoxin (BTx, magenta, asterisk) and myelin basic protein (MBP, magenta). D) Perineural recordings from distal motor nerves were performed on tissue from *Neuronal* and G*lial* mice treated with anti-GM1 Ab only or a source of complement (normal human serum, NHS) only. The peak of Na^+ and K^+ waveforms were measured and the ratio calculated. There were no differences between genotypes or treatments. Results are represented as the mean ± SEM, n=4/genotype/treatment, 5-10 recordings/mouse were analysed.

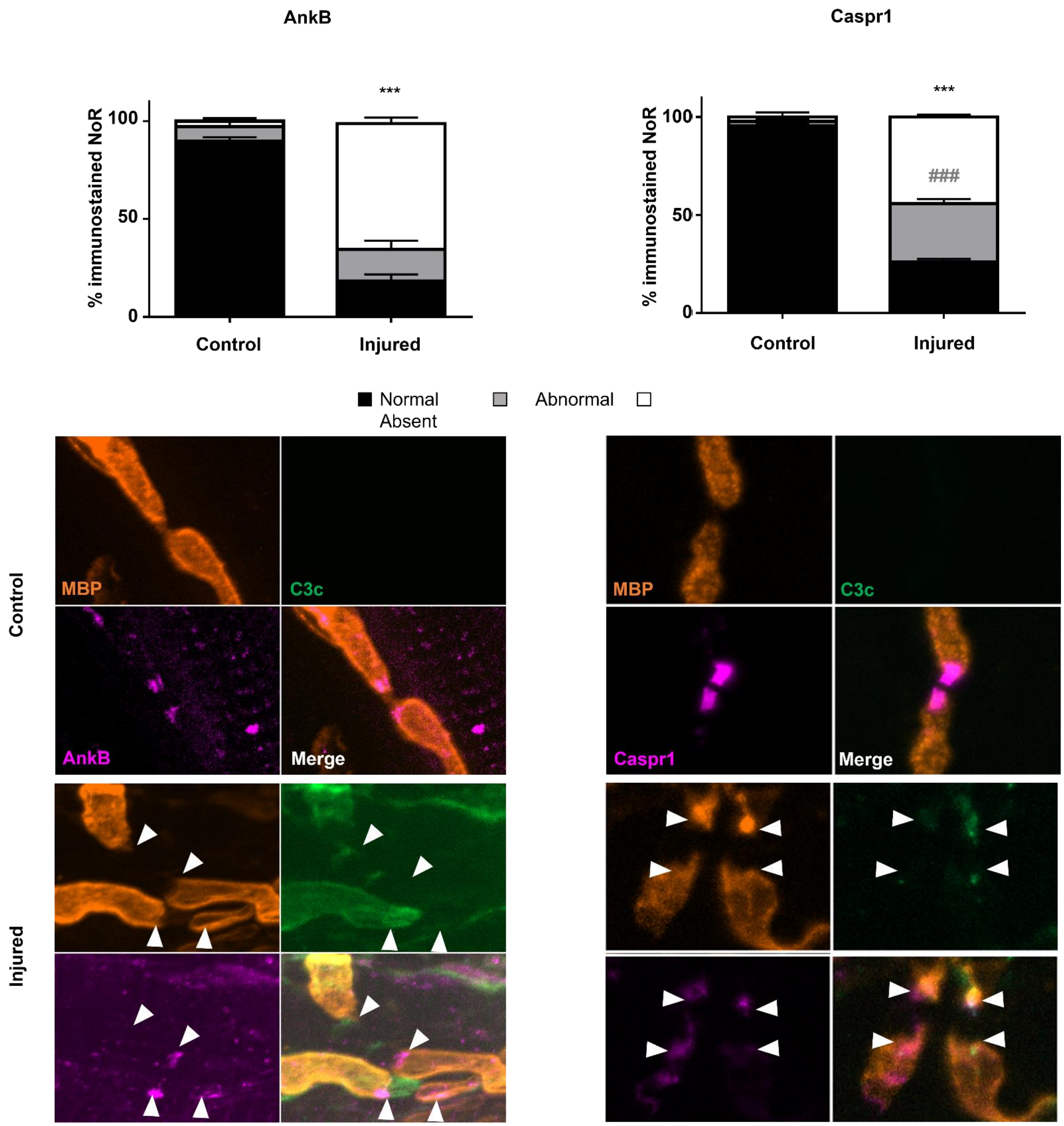

Supplemental Figure 4. Targeting the myelinating glia in WT mice with an anti-sulfatide antibody leads to the same paranodal disruption as anti-GM1 antibody in *Glial* tissue ex vivo. Triangularis sterni nerve-muscle preparations from wild type mice were treated ex vivo with anti-sulfatide Ab and a source of complement (injury, Inj) or anti-sulfatide Ab alone (control, Con) for 4h at 32°C. Disruption at the node of Ranvier (NoR) due to injury was assessed. NoR were identified by a gap in the myelin basic protein (MBP, orange) immunostaining and the site of Ankyrin B (AnkB) or Caspr1 (magenta) staining indicated by arrowheads. The presence of AnkB and Caspr1 (*, black bars) was significantly lower at injured NoR compared to control. Caspr1 was disrupted (#, grey bars) in injured tissue compared to control. Representative images show normal and absent/abnormal staining. Scale bar = 5 µm. Results are represented as the mean $\pm$ SEM, n=3/genotype/treatment, 15-30 NoR/mouse were analysed**.** Two-way ANOVA, * signifies p<0.05, ** signifies p<0.01 and *** signifies p<0.001.Two-way ANOVA, *** signifies p<0.001.

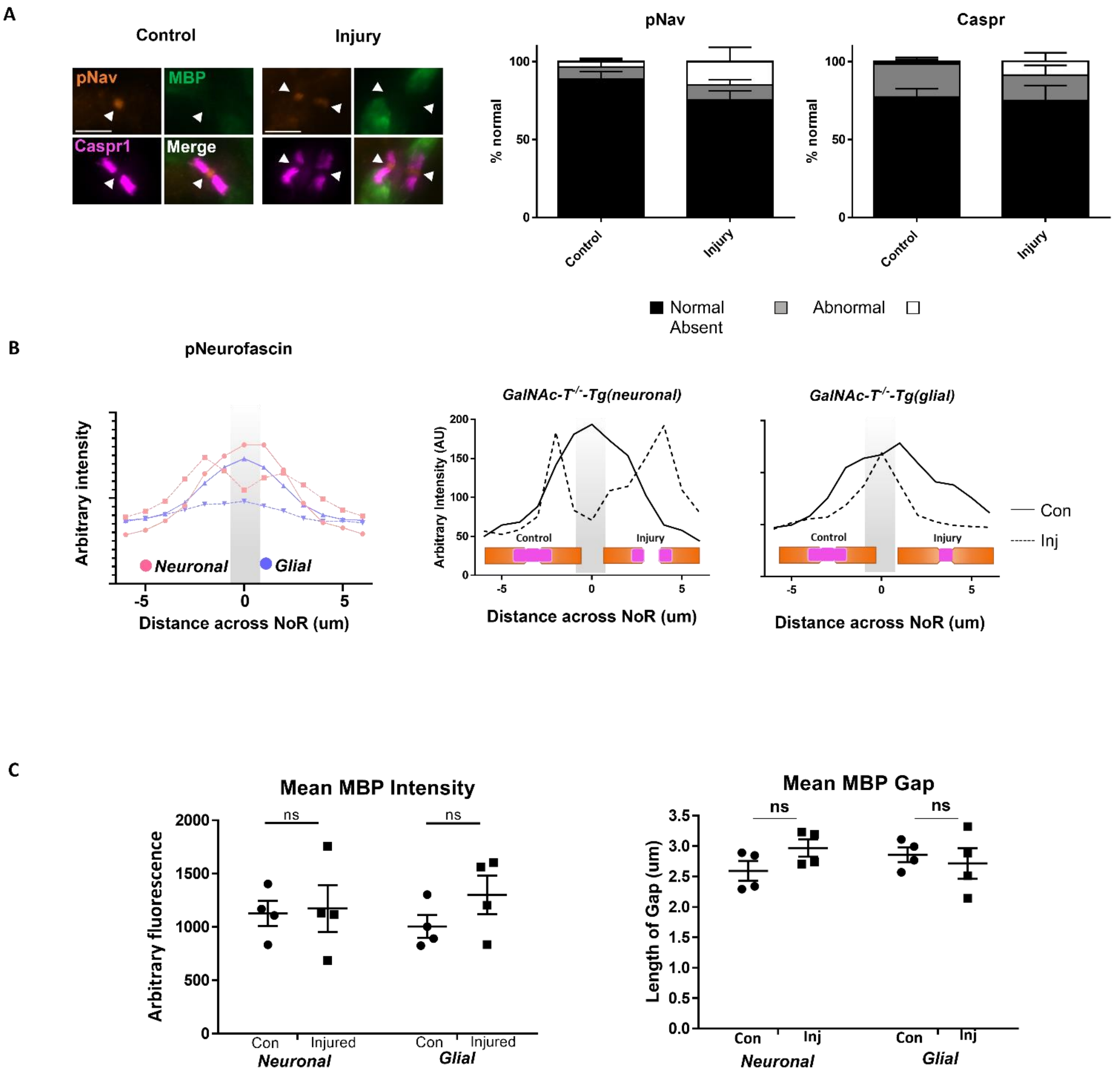

Supplementary Figure 5: Nav channel, Caspr1, neurofascin and myelin integrity following *Neuronal* and *Glial* membranes injury in vivo. Wild type (WT, A) or *Neuronal* and *Glial* (B & C) mice were dosed i.p. with 50 mg/kg anti-GM1 antibody followed 16 h later with 30 µl/g normal human serum (NHS)(Injury, Inj). Control mice were treated with the equivalent volume of PBS and NHS (control, Con). A) WT mice from the injury group had normal Nav channel (orange) and Caspr1 (magenta) immunostaining compared to control and were thus eliminated from further study. Scale bar = 5µm. B) The intensity of pan-neurofascin (NF) immunostaining across the node of Ranvier (NoR) centered on the nodal gap (grey shading) for each genotype was studied. The staining configuration in *Glial* mice was a single peak at the nodal gap, demonstrating a loss of glial paranodal NF155 and maintenance of axonal nodal NF186, while *Neuronal* traces revealed two intact lateral paranodal NF155 peaks and a loss of central nodal NF186 staining. C) MBP intensity along the distal internode among treatment groups did not change, suggesting myelin remains largely intact at this acute time-point. The gap between the myelin sheath, indicative of possible node of Ranvier lengthening, increased in injured *Neuronal* mice compared to all other treatment groups but did not reach significance. Results are represented as the mean + SEM, n=4/genotype/treatment. 15-30 NoR per mouse were analysed per mouse.

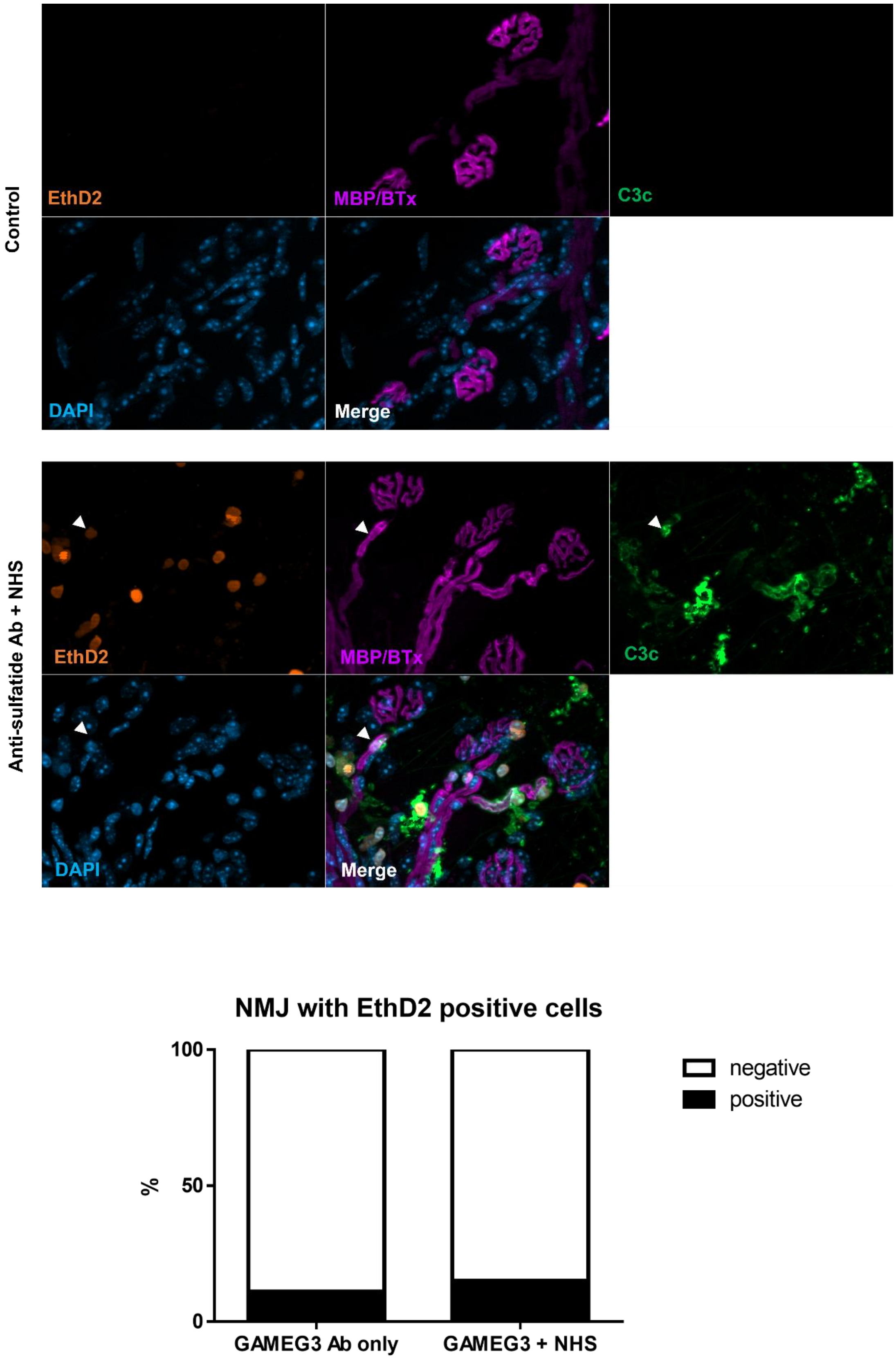

Supplementary Figure 6. Injury to non-myelinating perisynaptic Schwann cells does not contribute to secondary axon loss. Triangularis sterni nerve-muscle preparations from wild type (WT) were treated ex vivo with anti-sulfatide antibody and a source of complement (injury) or Ab alone (control) for 4 h at 32°C. There was no increase in motor nerve terminal (MNT)(magenta) number overlaid with ethidium homodimer (EthD-2, orange) positive cells in injured tissue compared to control. Representative images show the presence of complement deposition (green) in injured tissue and EthD-2 positive Schwann cell nuclei (orange) along nerve fibres. Results are represented as the mean ± SEM, n=3/treatment, 30-50 distal nerves per mouse were analysed. A student's t-test was used to determine statistical significance.
